# A novel SOD1-ALS mutation separates central and peripheral effects of mutant SOD1 toxicity

**DOI:** 10.1093/hmg/ddu605

**Published:** 2014-12-02

**Authors:** Peter I. Joyce, Philip Mcgoldrick, Rachele A. Saccon, William Weber, Pietro Fratta, Steven J. West, Ning Zhu, Sarah Carter, Vinaya Phatak, Michelle Stewart, Michelle Simon, Saumya Kumar, Ines Heise, Virginie Bros-Facer, James Dick, Silvia Corrochano, Macdonnell J. Stanford, Tu Vinh Luong, Patrick M. Nolan, Timothy Meyer, Sebastian Brandner, David L.H. Bennett, P. Hande Ozdinler, Linda Greensmith, Elizabeth M.C. Fisher, Abraham Acevedo-Arozena

**Affiliations:** 1 MRC Mammalian Genetics Unit, Harwell, OxfordshireOX11 0RD, UK,; 2 MRC Centre for Neuromuscular Disease, UCL Institute of Neurology, Queen Square, LondonWC1N 3BG, UK,; 3 Department of Neurology, Northwestern University, Feinberg School of Medicine, Chicago, IL 60611, USA,; 4 Nuffield Department of Clinical Neurosciences, University of Oxford, OxfordOX3 9DU, UK,; 5 Department of Cellular Pathology, Royal Free London NHS Foundation Trust, Pond Street, LondonNW3 2QG, UK; 6 UCL Cancer Institute, Paul O'Gorman Building, 72 Huntley Street, LondonWC1E 6BT, UK

## Abstract

Transgenic mouse models expressing mutant *superoxide dismutase 1* (*SOD1*) have been critical in furthering our understanding of amyotrophic lateral sclerosis (ALS). However, such models generally overexpress the mutant protein, which may give rise to phenotypes not directly relevant to the disorder. Here, we have analysed a novel mouse model that has a point mutation in the endogenous mouse *Sod1* gene; this mutation is identical to a pathological change in human familial ALS (fALS) which results in a D83G change in SOD1 protein. Homozgous *Sod1^D83G/D83G^* mice develop progressive degeneration of lower (LMN) and upper motor neurons, likely due to the same unknown toxic gain of function as occurs in human fALS cases, but intriguingly LMN cell death appears to stop in early adulthood and the mice do not become paralyzed. The D83 residue coordinates zinc binding, and the D83G mutation results in loss of dismutase activity and SOD1 protein instability. As a result, *Sod1^D83G/D83G^* mice also phenocopy the distal axonopathy and hepatocellular carcinoma found in *Sod1* null mice (*Sod1*^−/−^). These unique mice allow us to further our understanding of ALS by separating the central motor neuron body degeneration and the peripheral effects from a fALS mutation expressed at endogenous levels.

## Introduction

Amyotrophic lateral sclerosis (ALS) is a devastating neurodegenerative disease characterized by a loss of upper and lower motor neurons (LMNs), which causes muscle weakness, paralysis and ultimately death, typically within 3–5 years of disease onset. Approximately 10% of ALS cases have a clear family history (fALS), caused by mutations in specific genes, usually with a dominant pattern of inheritance (1–3). Mutations in *superoxide dismutase 1* (*SOD1*) account for 10–20% of fALS cases (although pathogenicity has not be demonstrated for all these changes), and to date >155 mutations have been identified throughout all five exons of the *SOD1* gene, >95% of which are dominant (4).

SOD1 is a 153 amino acid metalloenzyme (also called Cu/Zn SOD1) that forms a homodimer whose main known function is to remove superoxide radicals through creating molecular oxygen and hydrogen peroxide, although other functions are known (5). Mutant SOD1 takes on a toxic gain of unknown function in SOD1-fALS, causing many cellular abnormalities that ultimately result in death of motor neurons (6). Recent research has identified misfolded wild-type (WT) SOD1 in non-SOD1-fALS and in ‘sporadic’ ALS suggesting that it may also play a role in the pathogenesis of these ALS cases (7–10).

SOD1 is highly conserved across species (11) and >12 different transgenic mouse models have been created that overexpress mutant forms of human *SOD1* (6,12,13) and in one case mouse *Sod1* (14). The majority of these mice recapitulate many characteristics of ALS, including progressive motor deficits, hindlimb paralysis, motor neuron degeneration and early death (6,12,13). A mouse strain carrying a spontaneous point mutation in mouse *Sod1* has been previously described (15), although the equivalent mutation in humans has not been identified as pathogenic.

However, concerns remain regarding the translation of these models to human SOD1-fALS—particularly because SOD1 is generally overexpressed in transgenics and such raised expression levels affect the pathology of these animals (6,12). For example, the most widely used model of SOD1-fALS, the high-copy *SOD1^G93A^* transgenic, carries ∼24 copies of the mutant human *SOD1* gene, expresses SOD1 protein at ∼17-fold over the endogenous level, and has greatly accelerated disease compared with *SOD1^G93Adl^* mice, a strain derived from the *SOD1^G93A^* founder line but with lower levels of SOD1 protein because of a deletion in the transgene array (∼8–10 copies of *SOD1^G93A^* gene, 8-fold SOD1 protein expression over WT) (16–18). As well as raised levels of mutant SOD1 affecting phenotype, increased levels of WT SOD1 also give rise to neurodegeneration—overexpression of WT human SOD1 at levels comparable with that found in *SOD1^G93A^* transgenics results in an ALS-like syndrome with progressive loss of spinal motor neurons and premature death (19). Thus SOD1 ‘dose’ is clearly important for determining phenotype—and as well as overexpression, reduced expression also gives rise to neuronal and non-neuronal phenotypes in heterozygous and homozygous *SOD1* knockout mice (reviewed in 4).

Mutations in SOD1-ALS cause a toxic gain of function, which leads to motor neuron degeneration. However, curiously, the majority of studies that have analysed dismutase activity of *SOD1-*fALS patient samples show that SOD1 dismutase activity is reduced to an average of ∼58% of normal levels (reviewed in 4). *SOD1* transgenic models overexpress mutant SOD1 and also express two copies of endogenous mouse *Sod1*, so dismutase activity is not reduced in these animals. Therefore, although *SOD1* transgenics clearly model the SOD1 toxic gain-of-function leading to motor neuron degeneration, they do not generally model the possible effects on ALS pathogenesis of a reduction in dismutase activity, as observed in the majority of *SOD1*-fALS patient samples.

The effects of SOD1 loss of function on motor neurons have been recently readdressed through the study of *Sod1*^−/−^ mice (20,21), which suffer from a severe progressive denervation of hindlimb muscles, leading to striking motor phenotypes (20). Importantly however, several reports show that aged *Sod1*^−/−^ mice do not develop motor neuron degeneration at any age (20,22,23). Thus, SOD1 activity is critical in maintaining innervation of neuromuscular junctions, but its removal does not result in motor neuron cell body loss.

In order to create the genetically closest model of SOD1 ALS to date, and to investigate the effects of a *SOD1* mutation at endogenous expression levels, we identified a mutant line that carries an *N-*ethyl-*N*-nitrosourea (ENU)-induced point mutation in the mouse *Sod1* gene. Fortuitously, this mutation is identical to the nucleotide change found in human SOD1 D83G dominant fALS cases (24). In a D83G SOD1-fALS family, four of the five affected individuals had a rapid disease duration (range: 6–12 months), whilst one family member had a long disease duration (151 months). Two of the affected SOD1 D83G family members who were clinically examined in detail first presented with symptoms of LMN deficits, which were followed with upper motor neuron (UMN) symptoms (24).

## Results

### Identification of an ENU-induced point mutation in the mouse *Sod1* gene

To identify mouse lines carrying the equivalent of human ALS causative pathogenic mutations, we screened for mutations in *Sod1* using genomic DNA from an ENU-induced mutagenesis archive containing over 10 000 mice (25,26). We identified a mouse mutant carrying an adenosine-to-guanine missense mutation resulting in a D83G substitution (Supplementary Material, Fig. S1). Importantly, the same point mutation (A–G) gives rise to dominant D83G SOD1-fALS (24).

For all subsequent studies, female and male mice carrying the *Sod1^D83G^* mutation were assessed on a C57BL/6J genetic background, backcrossed at least four generations and then intercrossed. Homozygous *Sod1^D83G/D83G^* mice were not produced in Mendelian ratios from *Sod1^+/D83G^* intercrosses (in total: 167 WT, 362 *Sod1^+/D83G^*, 101 *Sod1^D83G/D83G^*) (Supplementary Material, Table S1).

### Upper and LMNs die in *Sod1^D83G/D83G^* mice

**Figure 1.**
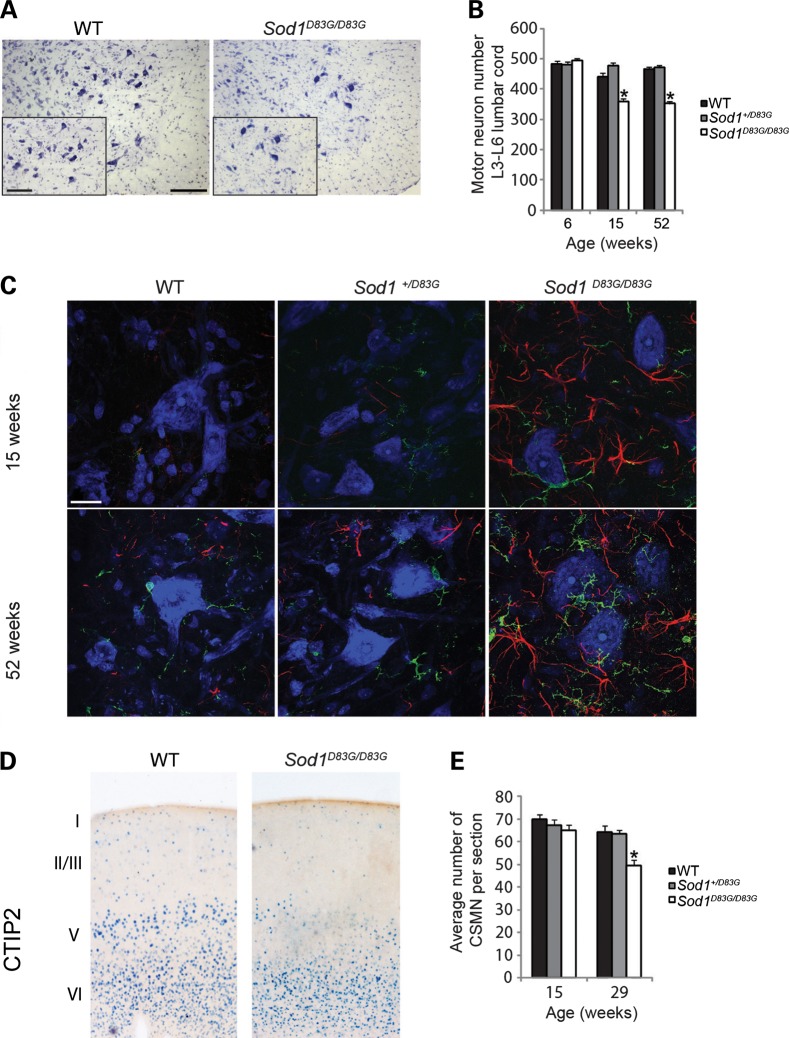
The *Sod1^D83G^* mutation causes LMN and UMN degeneration. (**A**) Representative images of lumbar spinal cord ventral horn sections stained for Nissl from WT and 15-week-old Sod1^D83G/D83G^ mice; sciatic pool of motor neurons depicted in inset image. Scale bars: main 200 μm, inset 100 μm. (**B**) LMN numbers at 6, 15 and 52 weeks of age in female littermates. At 6 weeks of age counts are comparable, but by 15 weeks of age in *Sod1^D83G/D83G^* have a 23% loss of LMNs (359 ± 9) compared with WT littermates (469 ± 11) and *Sod1^+/D83G^* (477 ± 11). Motor neuron survival of *Sod1^D83G/D83G^* mice (353 ± 9) at 52 weeks is not significantly reduced compared with that at 15 weeks (359 ± 9; *P* = 0.47). *n* ≥ 5 animals per group (*P < 0.001). (**C**) Representative images of ventral horn of lumbar spinal cord from 15- to 52-week-old mice stained for IBA-1 (green), GFAP (red) and Nissl (blue). Immunoreactivity for micro- and astrogliosis is increased in 15-week-old Sod1^D83G/D83G^ mice, and yet further in 52-week-old Sod1^D83G/D83G^ mice compared with WT littermates. Scale bar is 20 μm. (**D**) CTIP2 expression is detected in the striatum, in layer VI, and is restricted to CSMN within layer V of the motor cortex. (**E**) CSMN survival of female mice at 15 and 29 weeks of age. CSMNs are reduced in *Sod1^D83G/D83G^* mice at 29 weeks (49.7 ± 2.4) compared with WT littermates (64.1 ± 2.8). CSMNs within layer V were averaged across three slides per animal; *n* = 5 per group. Scale bar is 50 μm. Numbers represent the mean ± SEM (*P < 0.01).

Since degeneration of both LMN and UMNs is the defining hallmark of ALS and occurs in fALS patients carrying the D83G mutation (24), we first examined the survival of LMN and UMNs. The number of LMN in the sciatic motor pool in lumbar spinal cord was assessed at 6, 15 and 52 weeks of age. At 6 weeks, we found no loss of motor neurons in heterozygous *Sod1^+/D83G^* or homozygous *Sod1^D83G/D83G^* mice (WT 483 ± 12 LMN; *Sod1^+/D83G^* 481 ± 10 LMN; *Sod1^D83G/D83G^* 495 ± 8 LMN; *n* ≥ 5 per genotype). However, by 15 weeks there was a 23% reduction in the number of LMNs in *Sod1^D83G/D83G^* mice only (359 ± 9 LMN) compared with WT littermates (442 ± 11 LMN; *P* < 0.001) (Fig. 1A and B), and this remained stable at 52 weeks (Fig. 1B). Thus, homozygous *Sod1^D83G/D83G^* mice develop significant LMN degeneration between 6 and 15 weeks of age, which does not progress.

Reactive gliosis of both astrocytes and microglia is observed in ALS patients and mouse models. In *Sod1^D83G/D83G^* mice, lumbar spinal cord sections from 15-week-old mice showed striking astrogliosis (GFAP) and microgliosis (IBA1), which increased further at 52 weeks of age (Fig. 1C). *Sod1^+/D83G^* mice did not show elevation in reactive gliosis compared with WT littermates (Fig. 1C).

To investigate UMNs in *Sod1^D83G/D83G^* mice, we examined the survival of corticospinal motor neurons (CSMNs) at 15 and 29 weeks of age (Fig. 1D and E). Although Nissl staining of the cerebral cortex did not reveal obvious abnormalities in *Sod1^D83G/D83G^* mice at either age (Supplementary Material, Figure S2A), analysis of Cry*-*mu, a selective marker of CSMNs in layer V of the motor cortex (27), revealed signs of cellular degeneration in *Sod1^D83G/D83G^* mice at 29 weeks (Supplementary Material, Figure S2B). This was supported by staining for the molecular marker chicken ovalbumin upstream promoter transcription factor-interacting protein 2 (CTIP2), a transcription factor expressed by CSMN in layer V of the motor cortex and a subset of layer VI neurons (27) (Fig. 1D). CTIP2 expression helps distinguish between a possible reduction in molecular marker expression and cellular degeneration, and is restricted to the nucleus, allowing for reliable quantitative analysis.

CSMN numbers were comparable between WT, *Sod1^+/D83G^* and *Sod1^D83G/D83G^* littermates at 15 weeks of age (WT 70 ± 4; *Sod1^+/D83G^* 67 ± 5; *Sod1^D83G/D83G^* 65 ± 5) (Fig. 1E), but reduced by 22% at 29 weeks in *Sod1^D83G/D83G^* mice (WT 64 ± 6; *Sod1^+/D83G^* 64 ± 4; *Sod1^D83G/D83G^* 50 ± 5; *P* < 0.05) (Fig. 1D and E). This degeneration was restricted to CSMNs since analysis of callosal projection neurons (CPNs), which are developmentally closely related to CSMN but less vulnerable in ALS, showed that staining with CPN-specific markers LIM domain only four (LMO4) and special AT-rich sequence-binding protein 2 (SATB2), did not differ between WT and *Sod1^D83G/D83G^* littermates, at either age (Supplementary Material, Fig. S2C and D). These results therefore suggest a selective and progressive CSMN degeneration (UMN) within the cortical component of motor neuron circuitry, which is likely to affect the motor capability of *Sod1^D83G/D83G^* mice.

### Analysis of functional motor units reveals a distal motor neuropathy in *Sod1^D83G/D83G^* mice

**Figure 2.**
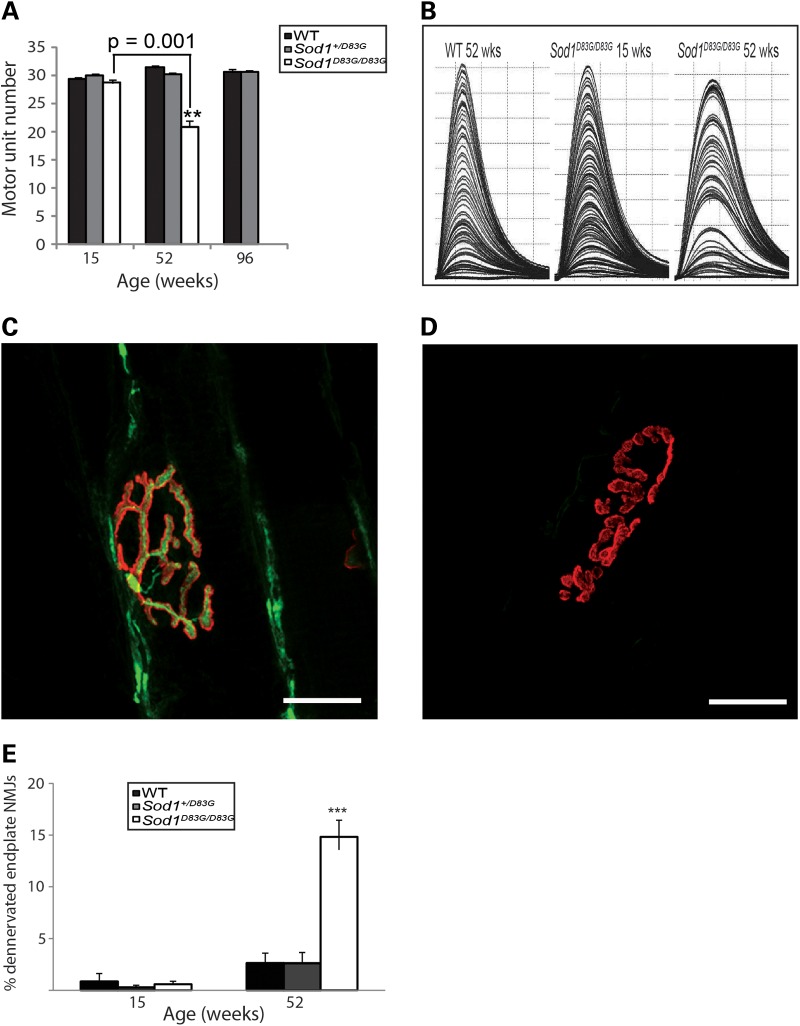
MUNE and endplate NMJ analysis from EDL muscle. (**A** and **B**) Surviving motor units for the EDL, which are reduced in 52-week-old *Sod1^D83G/D83G^* mice (21 ± 1) compared with WT littermates (39 ± 0.4). (B) Representative traces from (A); each twitch trace recording is a single motor unit. Numbers represent the mean ± SEM, at least nine legs were assessed per group. (**C**–**E**) Percentage of denervated endplate NMJ from EDL muscle at 15 and 52 weeks of age. Motor endplates are identified via α-bungarotoxin staining (red). Axons are revealed via neurofilament and SV2 (green). Representative images of (C) innervated and (D) denervated NMJ endplates. (E) Quantitative analysis of the percentage of denervated EDL endplate NMJ (denervated NMJ/total NMJ counted × 100) from all three genotypes at 15 weeks of age reveal no significant differences between any of the genotypes. By 52 weeks of age, a significant difference in the percentage of denervated endplate NMJ appear between *Sod1^D83G/D83G^* and the other two genotypes (WT and *Sod1^+/D83G^*). At 15 weeks of age, at least 700 NMJ endplates were counted per genotype. At 52 weeks of age, at least 465 NMJ endplates were counted per genotype. Percentage of denervated endplate NMJ at 52 weeks: WT: 2.6 ± 0.9; *Sod1^+/D83G^*: 2.6 ± 1.1; *Sod1^D83G/D83G^*: 14.8 ± 2.8. ***P < 0.001. Scale bar is 20 μm.

In view of the MN degeneration observed in *Sod1^D83G/D83G^* mice, we next determined the number of functional motor neurons that innervated the extensor digitorum longus (EDL) hindlimb muscle by physiological analysis of motor unit survival (MUNE). We found no differences in EDL motor unit survival across genotypes at 15 weeks of age, suggesting that the loss of LMN cell bodies detected at this age is restricted to populations of motor neurons that innervate hindlimb muscles other than EDL. Indeed, EDL has been previously shown to be less vulnerable to disease in transgenic *SOD1* models (28). However, by 52 weeks of age, we observed a significant reduction in the number of motor units in EDL muscles of *Sod1^D83G/D83G^* mice compared with WT littermates (*P* < 0.001; Fig. 2A and B). Comparison between WT and *Sod1^+/D83G^* littermates did not reveal any significant differences in EDL motor units at 15, 52 or 96 weeks of age (Fig. 2A).

Morphological analysis of the innervation pattern of endplate neuromuscular junctions (NMJ) of the EDL muscle confirmed the distal progressive denervation occurring between 15 and 52 weeks of age in *Sod1^D83G/D83G^* mice (Fig. 2C–E). In agreement with the MUNE analysis, no significant differences in denervated EDL endplate NMJ were observed between any of the genotypes at 15 weeks of age (Fig. 2E). However, by 52 weeks of age, a significantly different proportion of endplate NMJ in *Sod1^D83G/D83G^* EDL is denervated (WT 2.6 ± 0.9; *Sod1^+/D83G^* 2.6 ± 1.1; *Sod1^D83G/D83G^* 14.8 ± 2.8; *P* < 0.001) (Fig. 2E). Since no additional LMN body degeneration occurs between 15 and 52 weeks of age in *Sod1^D83G/D83G^* mice, the progressive denervation and loss of EDL motor units are likely a peripheral neuropathy that is not the result of motor neuron death.

### *Sod1^D83G/D83G^* mice display progressive motor and behavioural deficits

We next examined whether the loss of motor neurons and progressive denervation in *Sod1^D83G/D83G^* mice was reflected in deficits in motor function. Longitudinal phenotypic characterization of WT, *Sod1^+/D83G^* and *Sod1^D83G/D83G^* littermates showed motor function of *Sod1^D83G/D83G^* mice deteriorates progressively with age, and these mice develop tremors, gait abnormalities and become severely kyphotic (Supplementary Material, Video S1).

**Figure 3.**
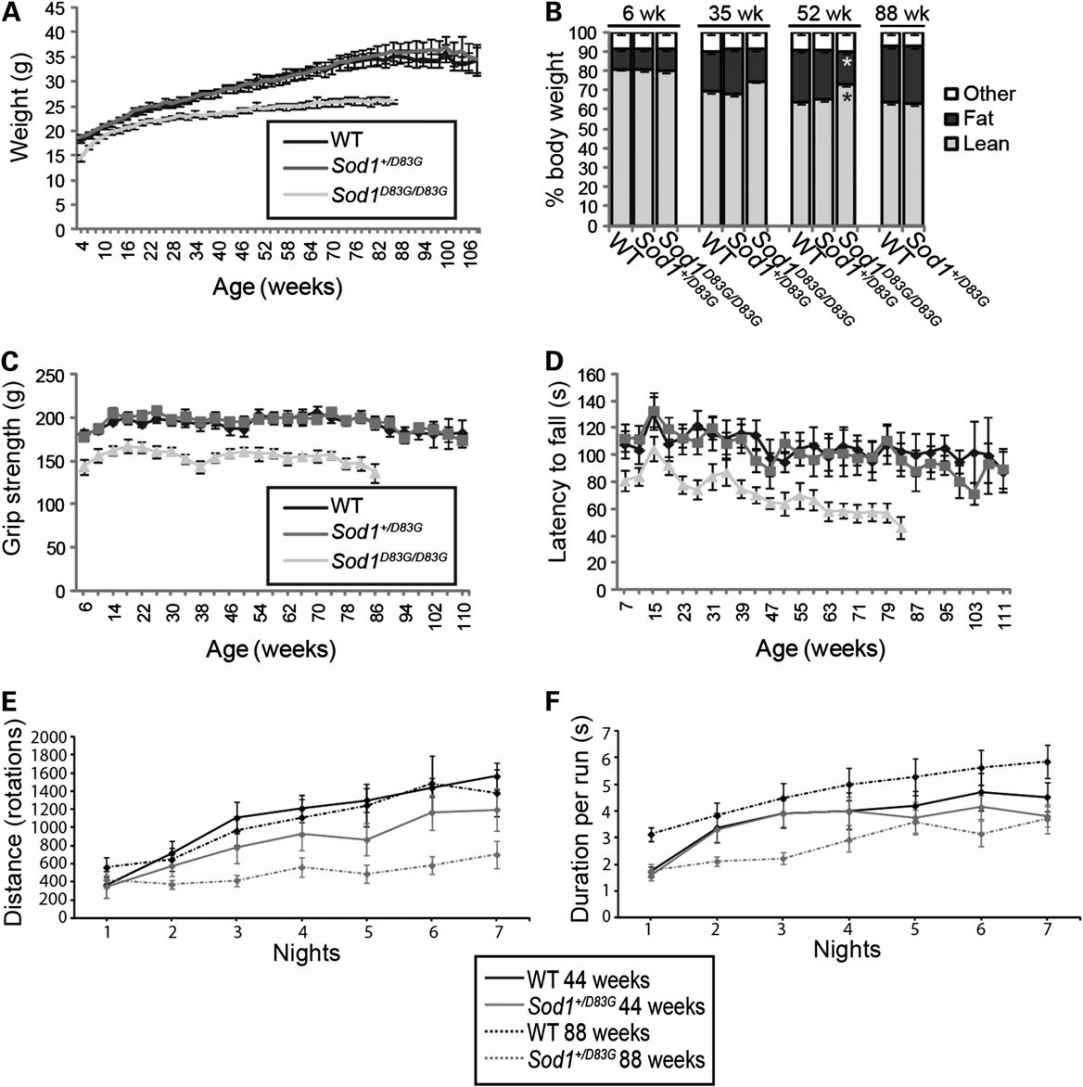
Body mass, behavioural and motor deficits in female *Sod1^D83G/D83G^* mice. (**A**) Weights recorded weekly from 4 weeks of age to the humane endpoint; cohort sizes for (A, **C** and **D**) started as 11 WT, 13 *Sod1^+/D83G^*, 11 *Sod1^D83G/D83G^*; due to the death of mice with age, at least five mice were assessed per genotype at later time points. Weight is reduced in *Sod1^D83G/D83G^* mice from 4 weeks of age (*P* = 0.001). (**B**) Echo MRI assessment of lean and fat mass for mice at 6, 35, 52 and 88 weeks of age. Fat mass is reduced and lean mass increased in 52-week-old *Sod1^D83G/D83G^* mice by comparison with WT littermates (*P ≤ 0.001). (C) Grip strength recorded monthly from 6 weeks of age to humane endpoint. Grip strength is reduced in *Sod1^D83/D83G^* mice (*P* ≤ 0.002 from 6 weeks). (D) Rotarod recorded monthly from 7 weeks of age to humane endpoint. Rotarod performance is reduced in *Sod1^D83G/D83G^* mice (*P* < 0.05 from 23 weeks). (**E** and **F**) Wheel running activity over 7 days from 44- and 88-week-old singly housed female WT and *Sod1^+/D83G^* littermates. The distance run (E) is shorter in 44-week-old *Sod1^+/D83G^* mice compared with WT (*P* < 0.01), while the distance run (E) and duration per run (F) both deteriorate with age in *Sod1^+/D83G^* mice (44 versus 88 weeks) compared with WT littermates (*P* < 0.05). *n* = 7 per genotype per time point. Numbers shown represent the mean ± SEM.

We found a reduction in body weight of *Sod1^D83G/D83G^* from 4 weeks of age compared with WT littermates (females, *P* = 0.001; males, *P* = 0.034) (Fig. 3A; Supplementary Material, Fig. S3A). To evaluate the weight differences between littermates, the relative levels of lean and fat mass were determined using Echo MRI (29). Fifty-two-week-old male and female *Sod1^D83G/D83G^* mice showed significantly less fat mass compared with sex-matched WT littermates, while there were no differences observed between WT and *Sod1^+/D83G^* littermates at 6, 35, 52 or 88 weeks of age (Fig. 3B; Supplementary Material, Fig. S3B).

To assess motor function, we examined grip strength and performance on an accelerating rotarod, starting at 6 and 7 weeks of age, respectively. We observed reduced grip strength in female and male *Sod1^D83G/D83G^* mice from 6 weeks of age (females, *P* ≤ 0.002; males, *P* < 0.05), but not in *Sod1^+/D83G^* mice (Fig. 3C; Supplementary Material, Fig. S3C). Female *Sod1^D83G/D83G^* mice displayed earlier deficits in accelerating rotarod compared with males (23 weeks versus 67 weeks) (Fig. 3D; Supplementary Material, Fig. S3D).

We also examined the performance of WT, *Sod1^+/D83G^* and *Sod1^D83G/D83G^* littermates using a modified SHIRPA analysis, which comprises of a battery of simple phenotypic tests with an emphasis on neurological function (30,31). Three traits differed between genotypes: the presence and progression of tremors, pelvic elevation and the ability to walk down a vertical wire grate (negative geotaxis) (Table 1).

**Table 1.** Semi-quantitative-modified SHIRPA phenotypic analysis of WT (*Sod1^+/+^*), *Sod1^+/D83G^* and *Sod1^D83G/D83G^* littermates

Genotype	Sample size	Mild tremors		Severe tremors		Reduced pelvic elevation		Negative geotaxis	
		Onset (weeks)	%	Onset (weeks)	%	Onset (weeks)	%	Onset (weeks)	%
*Sod1^+/+^* ♂	12	96	17	–	–	88	33	60	42
*Sod1^+/D83G^* ♂	15	94	13	–	–	86	20	69	60
*Sod1^D83G/D83G^* ♂	11	20	100	62	82	39	100	29	100
*Sod1^+/+^* ♀	13	98	15	–	–	86	46	90	8
*Sod1^+/D83G^* ♀	14	96	36	–	–	78	71	–	–
*Sod1^D83G/D83G^* ♀	12	22	100	67	58	32	100	49	75

Onset values are the mean age at onset, in weeks, for mice presenting each phenotype. Percentage represents the proportion of mice per line presenting each phenotype at least twice during their lifetime.

We found subtle behavioural deficits in heterozygous *Sod1^+/D83G^* animals when examining the performance of mice on an in-cage wheel running system (32). A similar system has previously been used to identify presymptomatic motor abnormalities in *SOD1^G93A^* transgenic mice (33). WT and *Sod1^+/D83G^* littermates were assessed at 44 and 88 weeks of age by recording activity over 7 days. *Sod1^+/D83G^* animals exhibited a significant deficit in nightly total distance travelled compared with WT littermates (*P* < 0.05), which declined further with age (Fig. 3E). In addition, while WT mice at 88 weeks of age showed an increase in the duration for each running bout (duration per run) when compared with 44 weeks of age, 88-week-old *Sod1^+/D83G^* mice showed a reduction in duration. *Sod1^+/D83G^* mice also displayed a reduction in the duration per running bout at 44 and 88 weeks of age compared with WT littermates (Fig. 3F). Thus, *Sod1^+/D83G^* mice present with subtle yet progressive deficits in motor function in the home cage, showing that the deleterious effects of the SOD1 D83G mutation are not restricted to homozygous mice.

### *Sod1^D83G/D83G^* mice lose muscle force with age

To gain a detailed understanding of the effect of the *Sod1^D83G^* mutation on motor neuron and muscle function, we undertook a physiological analysis of the tibialis anterior (TA) and EDL hindlimb muscles of female WT, *Sod1^+/D83G^* and *Sod1^D83G/D83G^* littermates at 15 and 52 weeks age. We also assessed *Sod1^+/D83G^* mice at 96 weeks of age.

**Figure 4.**
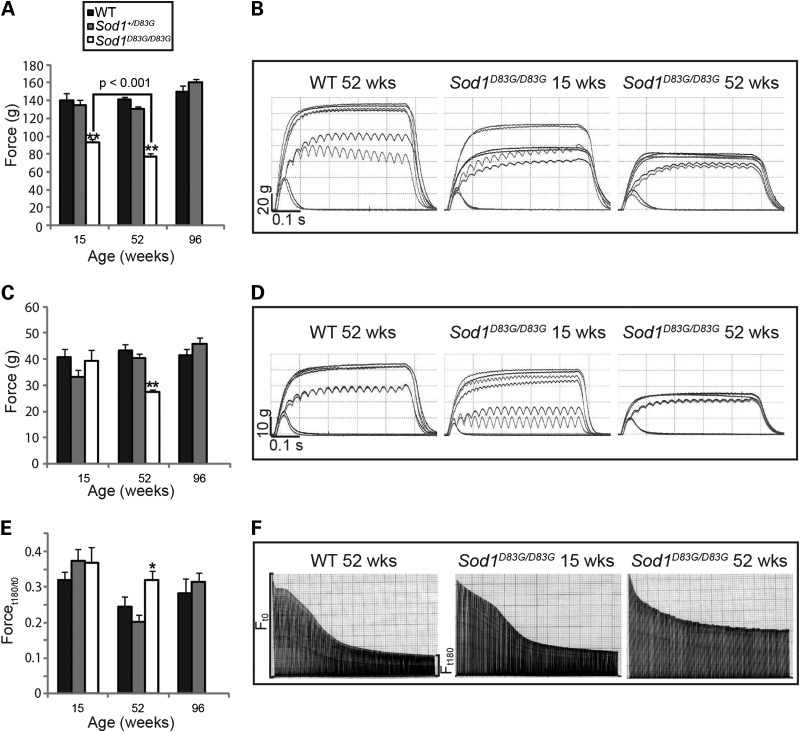
Functional deficits in muscle function in female *Sod1^D83G/D83G^* mice. (**A**) TA tetanic muscle force for mice at 15, 52 and 88 weeks of age; 15-week-old *Sod1^D83G/D83G^* TA tetanic muscle force (93 g ± 4 g) is reduced compared with WT littermates (140 ± 8 g) and deteriorates further at 52 weeks of age (77 ± 4 g). (**B**) Representative traces of TA tetanic tension from WT and *Sod1^D83G/D83G^* mice. (**C**) EDL tetanic muscle force: 52-week-old *Sod1^D83G/D83G^* EDL tetanic muscle force (29 ± 1 g) is reduced compared with WT littermates (43 ± 2 g). (**D**) Representative traces of EDL tetanic tension from WT and *Sod1^D83G/D83G^* mice. (**E**) FI of EDL muscle for mice at 15, 52 and 88 weeks of age. FI of 52-week-old *Sod1^D83G/D83G^* mice (0.32 ± 0.03) is increased compared with WT littermates (0.24 ± 0.03). (**F**) Representative fatigue traces from WT, and *Sod1^D83G/D83G^* mice, produced by repeated stimulation of the EDL muscle for 180 s. Each line in the trace represents a single tetanic tension; line length is proportional to force produced (see Materials and Methods). *P < 0.05, **P < 0.001.

TA muscle of 15-week-old *Sod1^D83G/D83G^* mice was significantly weaker than in WT littermates (*P* = 0.02), and muscle force declined further at 52 weeks of age (Fig. 4A and B). This may reflect the LMN degeneration detected in the spinal cord at this stage (Fig. 1B). However, as observed with motor unit survival and NMJ innervation, no deficit in EDL muscle force was detected in *Sod1^D83G/D83G^* mice at 15 weeks, confirming that this muscle is less vulnerable to the effects of mutant SOD1 than other hindlimb fast twitch muscles. However, by 52 weeks, EDL muscle force in *Sod1^D83G/D83G^* mice was significantly reduced by ∼35% compared with WT (*P* < 0.001; Fig. 4C and D). Therefore, TA and EDL muscles undergo a progressive loss in muscle strength in *Sod1^D83G/D83G^* animals between 15 and 52 weeks of age, at a time when there is no progression in the death of LMN. In addition, as with SOD1 transgenic mouse models, the TA is affected earlier than EDL (28).

TA and EDL are fast twitch muscles that normally fatigue rapidly when repeatedly stimulated. Changes in the fatigue characteristics of fast twitch muscles are a typical feature of disease in SOD1 transgenic mouse models (34) and so we undertook a fatigue test of EDL muscles in *Sod1^D83G/D83G^*mice (Fig. 4E and F). The force measured at the beginning of a 3 min period of stimulation was compared with that at the end of the test to produce a Fatigue Index (FI), a measure of the fatigability of the muscle. The results highlight the progressive deterioration of EDL in these mice, so that at 15 weeks, the FI of EDL in *Sod1^D83G/D83G^* mice is no different from WT, but by 52 weeks of age, EDL FI is increased by 30% in *Sod1^D83G/D83G^* mice compared with WT (*P* = 0.04) (Fig. 4E and F). Comparison between WT and *Sod1^+/D83G^* littermate mice did not reveal any significant differences in TA or EDL muscle function at 15, 52 or 96 weeks of age (Fig. 4).

### *Sod1^D83G/D83G^* mice have ALS-like changes in the histochemical phenotype of fast twitch muscles

To further characterize the effect of the *Sod1^D83G^* mutation on muscle physiology, we examined the oxidative capacity of the muscle fibres in the TA and EDL muscles, by staining for the oxidative enzyme succinate dehydrogenase (SDH) at 15 and 52 weeks of age. Even at 15 weeks of age, *Sod1^D83G/D83G^* TA muscle show a clear change in the histochemical properties of their muscle fibres (Supplementary Material, Fig. S4A and B). Fast twitch muscles, such as the TA and EDL, normally contain a large proportion of glycolytic fibres that stain only lightly for SDH activity, a marker of oxidative fibres. However, in the TA of *Sod1^D83G/D83G^* mice at 15 weeks of age, grouping of intensely stained type I slow oxidative muscle fibres was observed and by 52 weeks almost all of the muscle fibres in the TA of *Sod1^D83G/D83G^* mice stain intensely for SDH, indicating an oxidative phenotype that is more characteristic of slow twitch, type I muscle fibres (Supplementary Material, Fig. S4A). EDL muscles of 15-week-old *Sod1^D83G/D83G^* mice, had a similar SDH staining pattern to WT littermates, but by 52 weeks, there was a marked increase in the proportion of intensely stained, likely type I muscle fibres (Supplementary Material, Fig. S4B). These results once again indicate that in mice expressing mutant SOD1, EDL muscles are affected later in the disease than other hindlimb muscles such as the TA.

### *Sod1^D83G/D83G^* mice have selective sensory deficits

**Figure 5.**
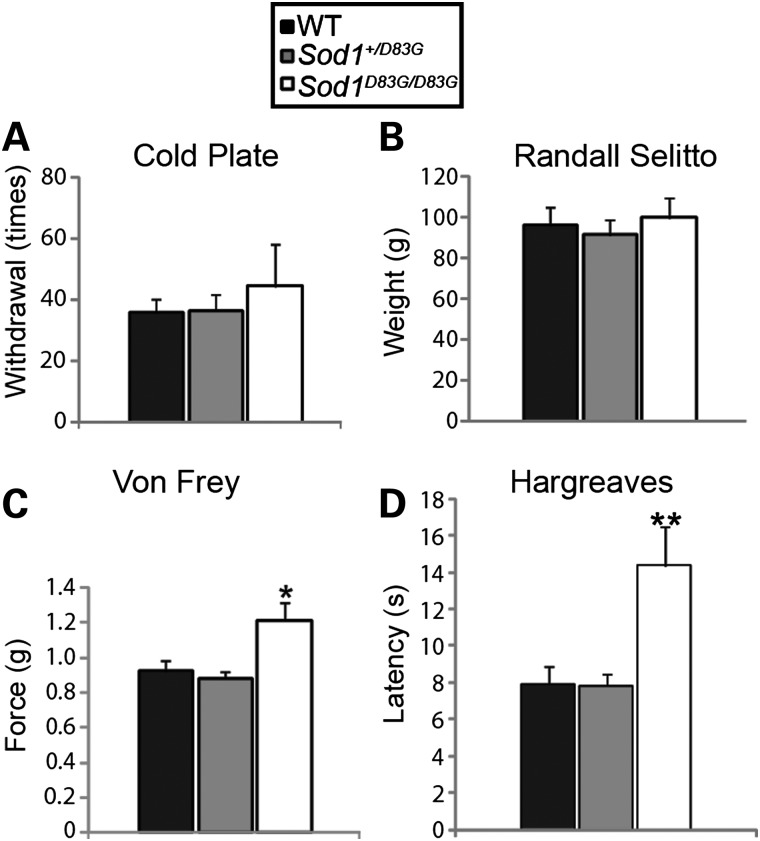
Behavioural sensory deficits in female *Sod1^D83G/D83G^* mice. No significant differences in cold nociceptive sensitivity (**A**, cold plate) or mechanical pressure threshold (**B**, Randall Sellito) between genotypes. (**C**) von Frey: *Sod1^D83G/D83G^* (1.22 g ± 0.10 g) display increased basal paw withdrawal threshold to von Frey filaments compared with WT littermates (0.92 g ± 0.06 g). (**D**) Hargreaves: *Sod1^D83G/D83G^* (14.3 s ± 2.1 s) display increased baseline heat withdrawal latency compared with WT littermates (7.9 ± 1.0 s). Numbers shown represent the mean ± SEM. At least 10 animals were assessed per genotype and time point (*P < 0.05, **P < 0.01).

Although ALS is predominantly a motor neuron disorder, a subset of ALS patients and *SOD1^G93A^* transgenic mice develop deficits in the sensory system (35,36). Thus, we performed behavioural sensory phenotypic analyses with four sensory tests on female mice at 22 weeks of age (*n* ≥ 10 per group): (i) cold plate (noxious cold stimulus), (ii) Randall Sellito test of mechanosensation (withdrawal to a high-threshold ramp mechanical stimulus), (iii) Von Frey (low threshold mechanical stimulus), (iv) Hargreaves method (noxious heat stimulus) (Fig. 5A–D). Interestingly, *Sod1^D83G/D83G^* mice have significant sensory deficits in response to both a low threshold mechanical stimulus (von Frey; *P* < 0.05) (Fig. 5C) and a noxious heat stimulus (Hargreaves method; *P* < 0.01) (Fig. 5D) but no deficit toward a noxious cold stimulus (cold plate) (Fig. 5A) or a high-threshold mechanical stimulus (Randall Sellito) (Fig. 5B). These results suggest that different primary afferent populations have selective vulnerabilities to the *Sod1^D83G^* mutation, or they may be a consequence of central sensorimotor integration.

### SOD1 D83G protein is dismutase inactive and at reduced levels in *Sod1^D83G/D83G^* mice

**Figure 6.**
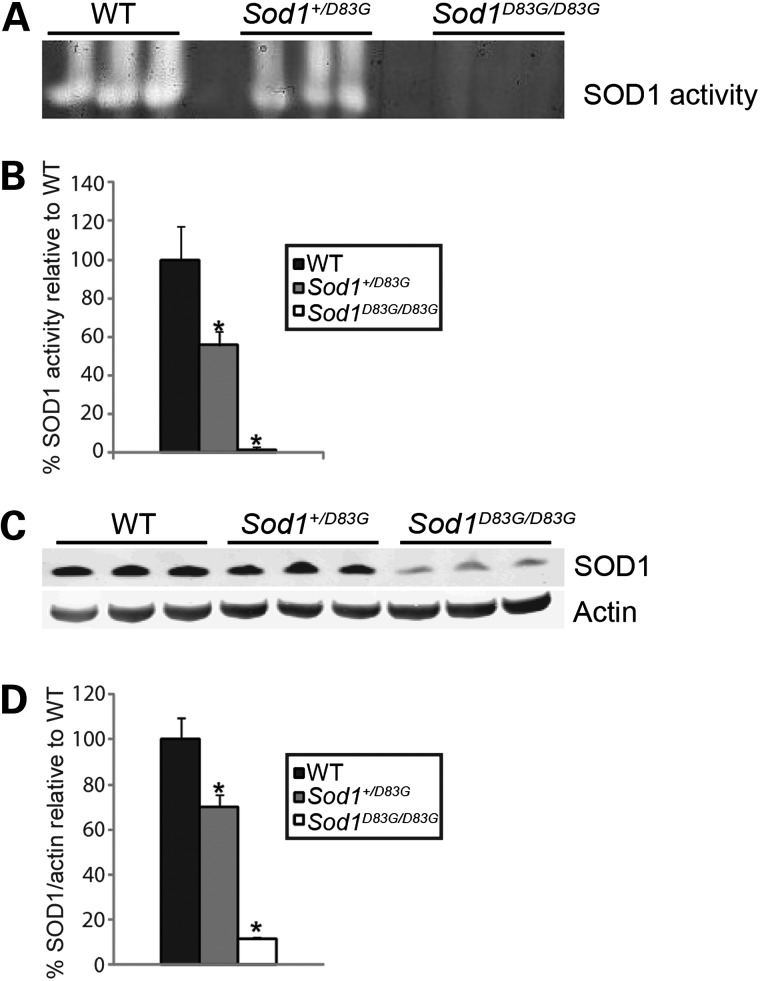
SOD1 D83G is dismutase inactive and unstably expressed. (**A** and **B**) SOD1 dismutase activity of brain homogenates from 65-week-old WT, *Sod1^+/D83G^* and *Sod1^D83G/D83G^* littermates, assessed using a nitroblue treazolium (NBT) in-gel assay. (A) Cleared areas indicate SOD activity. (B) Quantification of SOD1 dismutase activity across the three genotypes shows *Sod1^+/D83G^* (56% ± 7%) and *Sod1^D83G/D83G^* (1% ± 2%) homogenates have significantly less activity than WT (100% ± 17%) (*P = 0.002). (**C** and **D**) Immunoblot analysis of spinal cord soluble fractions for SOD1 protein levels from 65-week-old mice showing reduced SOD1 protein levels in *Sod1^+/D83G^* (70% ± 5%) and *Sod1^D83G/D83G^* (12 ± 0.4%) extracts compared with WT littermate extracts. Actin provides a protein loading reference; SOD1 levels are normalized to actin (*P < 0.001). Numbers represent the mean ± SEM. Values represent the average from three independent experiments.

The D83 residue coordinates zinc binding to SOD1, and is required for the correct folding of human SOD1 (37). Thus, mutating the D83 residue is likely to interfere with the correct folding of SOD1 and potentially affect SOD1 activity. We measured SOD1 dismutase activity from the brains of WT, *Sod1^+/D83G^*, and *Sod1^D83G/D83G^* littermates, and found *Sod1^D83G/D83G^* mice showed almost no SOD1 dismutase activity (1 ± 2%), whereas *Sod1^+/D83G^* brain homogenates have 56 ± 7% (*n* = 3 per genotype) of WT littermate SOD1 activity (Fig. 6A and B).

To assess if the D83G mutation affects SOD1 protein stability, we also measured protein levels of SOD1 in spinal cord from WT, *Sod1^+/D83G^* and *Sod1^D83G/D83G^* littermates. Intriguingly, SOD1 D83G protein levels are 12% ± 0.4% in *Sod1^D83G/D83G^* mice compared with 100% ± 9% in WT littermates, while in *Sod1^+/D83G^* mice SOD1 protein levels are intermediate at 70% ± 5% (*n* = 3 per genotype) (Fig. 6C and D).

The reduction of mutant SOD1 D83G in the soluble fraction might be due to an unfolding and sequestration to the insoluble fraction. Therefore, we measured the level of SOD1 in the RIPA-insoluble fractions from spinal cords of WT, *Sod1^+/D83G^* and *Sod1^D83G/D83G^* littermates and found low levels of insoluble SOD1 in all genotypes, with a similar reduction in SOD1 D83G protein levels in *Sod1^+/D83G^* and *Sod1^D83G/D83G^* mice as that observed in the soluble fraction (Supplementary Material, Fig. S5A). Thus, SOD1 D83G protein does not accumulate in the insoluble fraction.

We were unable to identify misfolded SOD1 by immunoprecipitation using either the SEDI or USOD misfolded SOD1 antibodies from *Sod1^+/D83G^* and *Sod1^D83G/D83G^* tissue extracts or immunohistochemistry (Supplementary Material, Figs. S5B and S6A), or detect overt inclusion pathology using p62 or ubiquitin staining in *Sod1^+/D83G^* and *Sod1^D83G/D83G^* mice from spinal cord and brain (Supplementary Material, Figs. S5C and S6B).

To examine whether *Sod1^D83G^* mRNA is differentially regulated from WT *Sod1*, we performed quantitative PCR of *Sod1* mRNA isolated from brains of 9-week-old WT, *Sod1^+/D83G^*, *Sod1^D83G/D83G^* littermates and did not observe a difference between genotypes (Supplementary Material, Fig. S7A). In addition, we directly compared the relative levels of *Sod1* WT and *D83G* mRNA levels from the brains of 9-week-old *Sod1^+/D83G^* mice using quantitative pyrosequencer analysis (see Materials and Methods) and did not see a difference between the relative expression levels of WT and mutant alleles (Supplementary Material, Fig. S7B).

### *Sod1^D83G/D83G^* have some phenotypes of *Sod1* null mice

Since *Sod1^D83G/D83G^* mice are dismutase inactive (Fig. 6A and B), they are likely to phenocopy at least some aspects of *Sod1* null mice (*Sod1^−/−^*). *Sod1^−/−^* mice develop a progressive peripheral motor neuropathy, in which motor axons retract from neuromuscular junctions resulting in muscle denervation (20,22). However, unlike *Sod1^D83G/D83G^* mice, there is no reported motor neuron loss in *Sod1^−/−^* mice at any age (20,22,23). To verify that LMN loss in *Sod1^D83G/D83G^* mice is not due to a loss of SOD1 activity and to confirm that motor neuron survival is unaffected in *Sod1^−/−^* mice, we compared LMN survival in female *Sod1^D83G/D83G^* and *Sod1^−/−^* mice at 15 weeks of age; both genotypes were backcrossed to a C57BL/6J background. In contrast to *Sod1^D83G/D83G^* mice and in agreement with all previous reports, *Sod1^−/−^* mice do not develop motor neuron cell body loss and the survival of motor neurons in the sciatic pool is similar in WT (484 ± 9 MN, *n* = 6) and *Sod1^−/−^*(489 ± 6 MN, *n* = 5; *P* = 0.47) mice. Therefore, unlike *Sod1^−/−^* mice, *Sod1^D83G/D83G^* mice have an additional toxic gain of function that causes the degeneration of motor neuron cell bodies.

**Figure 7.**
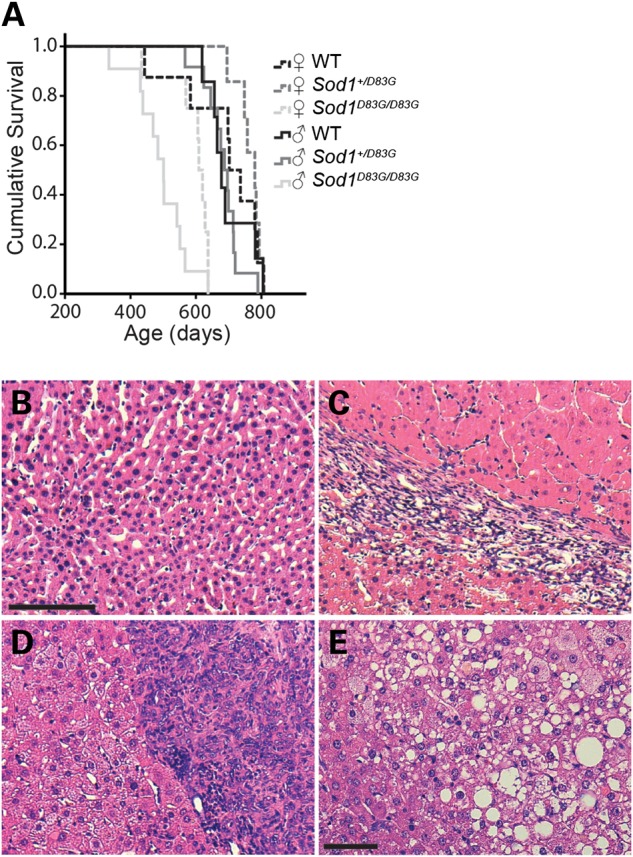
Lifespan of *Sod1^D83G/D83G^* mice and the presence of hepatocellular carcinoma. (**A**) Survival of male and female WT, *Sod1^+/D83G^* and *Sod1^D83G/D83G^* littermates to humane endpoint (see Materials and Methods). *Sod1^D83G/D83G^* mice reach end-stage sooner than WT and *Sod1^+/D83G^* littermates (Table 2) and male *Sod1^D83G/D83G^* mice (495 ± 22 days) reach end-stage earlier than females (588 ± 24 days) (*P* = 0.024). (**B**–**E**) Hematoxylin and eosin (H&E) stained liver sections from age-matched (B) WT and (C–E) end-stage *Sod1^D83G/D83G^* mice. Livers from *Sod1^D83G/D83G^* mice contain well-defined hepatocellular carcinoma (C) within an otherwise morphologically normal liver. (D) A poorly differentiated hepatocellular carcinoma or cholangiocarcinoma (E) within an otherwise inflamed liver background. Scale bar for (B)–(D) is 100 μm, and for (E) is 50 μm.

Lifespan is reduced in transgenic SOD1 mice that overexpress mutant SOD1 and in *Sod1^−/−^* mice. We set the humane endpoint of life in *Sod1^D83G/D83G^* mice as either the time at which a loss of 20% of maximum bodyweight occurs or the onset of piloerection (an involuntary erection of fur that is indicative of loss of health). The majority of WT, *Sod1^+/D83G^* and *Sod1^D83G/D83G^* littermates were culled due to a loss of 20% bodyweight (∼70% of *Sod1^D83G/D83G^* mice), while the rest (∼30%) were culled for the presence of piloerection. The survival of *Sod1^D83G/D83G^* mice is shortened compared with *Sod1^+/D83G^* and WT mice (Table 2 and Fig. 7A). We also found that male *Sod1^D83G/D83G^* mice had a significantly reduced lifespan compared with female *Sod1^D83G/D83G^* mice (495 ± 22 days versus 588 ± 24 days; *P* = 0.024).

**Table 2.** Incidence of hepatocarcinogenesis and average survival of *Sod1^D83G/D83G^* mice

Genotype	No. of animals examined		Average survival (days)		No. of animals with abnormal liver^a^	
	Males	Females	Males	Females	Males	Females
*Sod1^+/+^*	9	11	710 ± 21	754 ± 22	1 (11%)	0 (0%)
*Sod1^+/D83G^*	15	13	696 ± 14	779 ± 15	2 (13%)	0 (0%)
*Sod1^D83G/D83G^*	11	9	495 ± 22	588 ± 24	10 (91%)	7 (78%)

^a^Gross analysis of liver and presence of abnormal nodules.

At autopsy, *Sod1^D83G/D83G^* mice had significantly more liver tumours than WT and *Sod1^+/D83G^* littermates (Table 2; *P* < 0.001). Interestingly, it has previously been shown that *Sod1^−/−^* mice also develop liver tumours, most likely as a result of an increase in oxidative damage (38). Given that *Sod1^D83G/D83G^* mice are dismutase inactive for SOD1 (Fig. 6A and B), it is likely that the loss of SOD1 activity in these mice is the cause of liver tumours.

The identification of liver tumours in *Sod1^D83G/D83G^* mice led us to examine end-stage male *Sod1^D83G/D83G^* mouse livers. Livers from WT littermates had no pathology other than variable perivenular lymphocytic infiltration (Fig. 7B). In *Sod1^D83G/D83G^* mice, tumours occurred in the presence of otherwise pathologically normal and abnormal livers. In Figure 7C, there is a single, well-circumscribed and well-differentiated hepatocellular carcinoma occurring in a liver that is otherwise morphologically normal. In contrast, another *Sod1^D83G/D83G^* liver demonstrated multiple ill-defined hepatocellular carcinomas and a well-circumscribed nodular lesion, composed of sheets of pleomorphic tumour cells with vesicular nuclei and basophilic cytoplasm compatible with a poorly differentiated hepatocellular carcinoma or cholangiocarcinoma (Fig. 7D). This liver also showed diffuse nuclear anisocytosis, moderate parenchymal inflammation and patchy fatty change (Fig. 7E). Hence, pathological analysis reveals that the hepatocellular carcinomas in *Sod1^D83G/D83G^* mice occurred in the presence of both morphologically normal and abnormal livers.

### Mitochondrial membrane potential defects in *Sod1^+/D83G^* embryonic motor neurons

**Figure 8.**
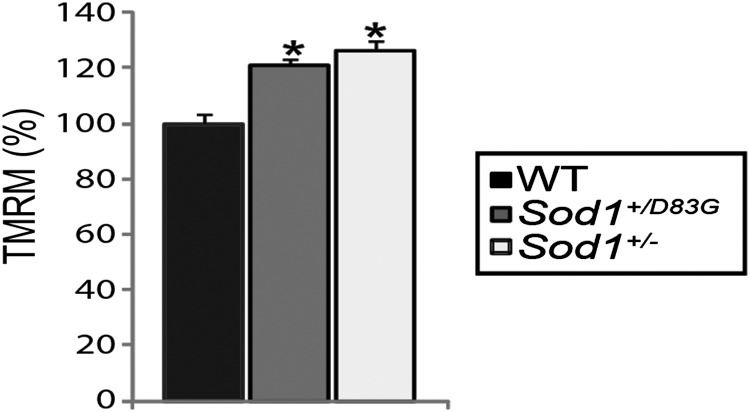
Mitochondrial potential in *Sod1^+/D83G^* and *Sod1*^+/−^ motor neurons. Resting Δ*ψ*_m_, estimated using TMRM, from embryonic WT littermates, *Sod1^+/D83G^* and *Sod1^+/−^* motor neurons (WT, 100% ± 3.4%; *Sod1^+/D83G^*, 121% ± 1.9%; *Sod1^+/−^*, 126% ± 3.8%). Data are normalized to resting Δ*ψ*_m_ for WT littermates (100%) and represent the mean ± SEM. Recordings taken from at least 75 cells from 2 or more independent embryonic motor neuron cultures (*P < 0.001).

Mitochondrial dysfunction has been identified as one of the earliest defects in motor neurons of transgenic SOD1 mice and it has been suggested that mitochondrial dysfunction may play a pivotal role in motor neuron degeneration in ALS (39). We therefore analysed the mitochondrial membrane potential (Δ*ψ*_m_) of embryonic motor neurons. Δ*ψ*_m_ is an indicator of mitochondrial energetic state and can be measured using tetramethylrhodamine methylester (TMRM) (40). Those from *Sod1^D83G/D83G^* (as previously reported for *Sod1^−/−^*) did not survive for >72 h in culture and therefore Δ*ψ*_m_ could only be examined in motor neurons from WT and *Sod1^+/D83G^* littermates. We have previously shown that Δ*ψ*_m_ in embryonic motor neurons from *SOD1^G93A^* transgenic mice is disrupted (41). In embryonic motor neurons of *Sod1^+/D83G^* mice, Δ*ψ*_m_ was significantly elevated compared with WT littermates (Fig. 8; *P* < 0.001).

Since the *Sod1^+/D83G^* mice have an ∼45% reduction in SOD1 dismutase activity, it was unclear whether the elevation in Δ*ψ*_m_ in *Sod1^+/D83G^* motor neurons was due to a loss of SOD1 dismutase activity, or a gain of function of SOD1 D83G protein. We therefore analysed the Δ*ψ*_m_ of embryonic motor neurons derived from WT and *Sod1^+/−^* littermates and found that *Sod1^+/−^* motor neurons also have elevated Δ*ψ*_m_ (Fig. 8). This indicates that the mitochondrial defects present in *Sod1^+/D83G^* motor neurons are likely caused by a partial loss of SOD1 function.

## Discussion

We have identified and characterized the first mouse model carrying the equivalent of a human ALS pathogenic mutation in the endogenous mouse *Sod1* gene, and this mutation is identical to a human fALS mutation (24). Since the mutation is within the endogenous *Sod1* gene, mutant SOD1 is not overexpressed. Here we show that homozygous *Sod1^D83G/D83G^* mice develop upper and LMN degeneration, unlike *Sod1* null animals. This is therefore likely due to a toxic gain of function of mutant SOD1—presumably modelling that which causes motor neuron death in human ALS cases.

We found ∼20% loss of UMN by 29 weeks of age in homozygotes, but did not look at later time points and so do not know if this phenotype progresses. Given the importance of mutant SOD1 protein dose in accelerating the ALS-like phenotype (16), it is possible heterozygotes may develop UMN loss later in life. Alternatively, a copy of WT SOD1 might be protective; this remains to be determined.

We showed 23% loss of LMN in the lumbar spinal cord of homozygous *Sod1^D83G/D83G^* mutants by 15 weeks of age that remained stable at 52 weeks of age. We speculate whether further ageing, or a ‘second hit’ might be required to cause a more dramatic loss of LMN in this mouse mutant; however, this remains to be determined. We note that a similar loss of MN in humans is unlikely to cause sufficient loss of motor function to prompt a visit to a physician. *Sod1^+/D83G^* heterozygous mutants displayed no LMN loss up to 52 weeks of age, either because the dose of mutant SOD1 was insufficient or because WT SOD1 might have a protective effect.

Our analyses of the *Sod1^D83G^* mouse mutant show that mutation in the endogenous *mouse Sod1* gene models critical features of human ALS (U and LMN cell death), albeit with a different profile than found in mice expressing human mutant *SOD1* transgene arrays. This corroborates earlier findings in which a mutant genomic mouse *Sod1* transgenic was used to model human SOD1 G85R-fALS—‘*Sod1* G86R’ mice with ‘high transgene expression’ had both U and LMN degeneration by 3–4 months of age (14). Therefore, the endogenous mouse SOD1 D83G mutation is informative for determining why some U and LMN die in ALS but removes any possible confounding effects of overexpression observed in SOD1 transgenic mice. We note that we and others have not observed MN cell body degeneration in *Sod1^−/−^* mice at any stage, and therefore MN loss in *Sod1^D83G/D83G^* mice likely arises from a toxic gain of function of the mutant SOD1 protein, as in ALS (20–22).

The significant reduction in SOD1 D83G protein is not due to allele-specific differences at the transcriptional level, but the dose-dependent decreases in SOD1 protein in heterozygous and homozygous D83G mutant mice may result from instability of mutant SOD1 and its subsequent degradation (42). It has been previously reported that mutant SOD1 has decreased half-life compared with WT SOD1 (42), and potentially the inability of D83G SOD1 to coordinate zinc may contribute to its instability (37,43,44).

Given that SOD1 expression levels in different transgenic mouse models show dose-dependent toxicity (16,18), the low levels of SOD1 D83G protein may only be sufficient to cause moderate motor neuron degeneration. The low SOD1 mutant protein level (∼10% of WT in homozygotes) may be sufficient to elicit a degree of motor neuron degeneration but not the levels of loss seen in ALS, and may at least partially explain why loss of motor neurons within the sciatic motor pool appears not to progress between 15 and 52 weeks of age.

With respect to loss-of-function effects, which are of interest because of the loss of dismutase activity in the majority of human pathogenic SOD1-fALS mutations (average dismutase activity in SOD1-fALS is ∼58% of normal (4)), we found mitochondrial membrane potentials (Δ*ψ*_m_) of both *Sod1^+/D83G^* and *Sod1^+/−^* embryonic MNs are hyperpolarized. Thus, SOD1 loss of dismutase function to just 56% of WT (*Sod1^+/D83G^* level) contributes to mitochondrial abnormalities. Distal axonopathy in *Sod1^−/−^* mice can be rescued by expressing mitochondrial-targeted WT SOD1 (20); the relevance of this to ALS remains to be determined.

*Sod1^D83G/D83G^* mice develop a severe peripheral neuropathy similar to that arising from the loss of function in *Sod1*^−/−^ mice. This peripheral neuropathy is likely driving the phenotypic deterioration from 15 weeks of age in *Sod1^D83G/D83G^* mice (Supplementary Material, video S1), as no further LMN loss occurs after that. As dismutase activity is only 1% of WT animals in the SOD1 D83G homozygotes, this peripheral neuropathy may be due to a loss of dismutase activity, potentially leading to increased vulnerability of peripheral motor axons to oxidative stress (45). It is also possible that the neuropathy develops because of a loss-of-unknown SOD1 function.

In at least one patient with diagnosed ALS who unexpectedly died from other causes, NMJ degeneration was found to precede motor neuron cell body death (45). Similarly, in transgenic human mutant SOD1 mouse models (with normal dismutase levels), NMJ degeneration leading to synaptic dysfunction precedes motor neuron cell body death and behavioural motor deficits (45,46). These findings led to the concept of ALS as a “dying back” disorder in which muscle denervation precedes the death of the motor neuron cell body, and suggests that SOD1 gain of function can also lead to NMJ degeneration—and potentially also contributing to the distal neuropathy seen in *Sod1^D83G/D83G^* mice.

Here our data, and that from other SOD1 mouse models, suggests that the axonal and neuronal cell body degeneration may be separate events that could be modulated by different sets of genes and/or environmental factors. In further support of this hypothesis, ablation of *Bax* in the human G93A SOD1 transgenic mouse model leads to complete dissociation between motor neuron soma death and motor dysfunction via distal denervation (47). While cell bodies are protected in *Bax* deficient G93A SOD1 transgenic mice, the degree of NMJ denervation and overall survival is not altered compared with G93A transgenic mice alone. Thus, *Sod1^D83G/D83G^* mice provide a novel model to test therapeutics aimed at preserving NMJ as well as ameliorating the early stages of motor neuron cell body degeneration.

Overall, these results suggest that *Sod1^D83G/D83G^* mice not only model the early stages of human ALS but are also able to separate the effects of central neuronal death from the peripheral distal neuropathy in a system in which the mutant gene is expressed at endogenous levels.

The *Sod1^D83G^* model is the first of its kind carrying a known pathogenic point mutation in the mouse endogenous *Sod1* gene, which is identical to a human fALS mutation. Homozygous *Sod1^D83G/D83G^* mice develop a degree of UMN and LMN degeneration, as well as progressive motor dysfunction due to a distal neuropathy, and hence model key aspects of the early stages of ALS. The *Sod1^D83G^* model therefore provides a unique mammalian system in which to assess the contribution of both central neuronal loss and peripheral axonal dysfunction, which will further our understanding of ALS.

## Materials and Methods

### Mice

*Ethics statement*: all experiments were performed under licence from the UK Home Office. WT, *Sod1^+/D83G^* and *Sod1^D83G/D83G^* mice were initially on a C57BL/6J-C3H background and backcrossed at least four generations to C57BL/6J. For all experiments, littermates for all genotypes were used, produced by intercrossing *Sod1^+/D83G^* mice. Experiments were performed blind to genotype and lifespan was defined as a loss of 20% of maximum body weight or the presence of piloerection. Animals were assessed daily and weighed at least biweekly.

### Identification of *Sod1^D83G^* mutation

The Harwell ENU-DNA archive (http://www.har.mrc.ac.uk/services/dna_archive/) was screened with high-resolution melting analysis using the LightScanner platform (Idaho Technology Inc., Salt Lake City, Utah, USA) (48). Coding sequence from exon 4 of *Sod1* including flanking (∼70 bp) splice sites was tested in DNA derived from ∼10 000 F1 ENU mutagenized animals. *Sod1* exon 4 was amplified with the following primers: SOD1_4F CATCCACTCATACGTATTTGAC, SOD1_4R ACCATAAAGTCATGGGAAGG using the lightscanner mastermix plus LC green (Idaho Technology Inc.) and possible mutations analysed with Sanger sequencing (GATC, Germany). We identified the chromosome 16 A90,224,530G mutation, corresponding to the D83G amino acid change in SOD1. The mouse line was rederived using sperm from the (C57BL/6J × C3H) F1 founder by *in vitro* fertilization. Genomic DNA from the F1 founder carrying the *Sod1 D83G* mutation was deep sequenced (see Supplementary Material, Materials and Methods for details) to identify any other possible ENU-induced mutations linked to *Sod1* on mouse chromosome 16. The closest ENU-induced coding mutation was ∼46 Mb proximal to the *Sod1* gene and did not segregate with lifespan or weight abnormalities in *Sod1^D83G/D83G^* mice.

### Behavioural analysis

See Supplementary Material, Materials and Methods for a description of behavioural and Echo MRI analysis.

### Physiological assessment of hindlimb muscle force, motor units and fatigue index

Muscle force, motor unit number and muscle fatigue characteristics for female WT, *Sod1^+/D83G^* and *Sod1^D83G/D83G^* littermates were examined at 15 and 52 weeks of age, and female WT and *Sod1^+/D83G^* littermates also at 88 weeks of age, as described previously (49). Cohort sizes per time point were at 15 weeks: 5 WT, 5 *Sod1^+/D83G^* and 5 *Sod1^D83G/D83G^* animals; 52-weeks: 5 WT, 7 *Sod1^+/D83G^* and 6 *Sod1^D83G/D83G^* animals; 88 weeks: 5 WT, 7 *Sod1^+/D83G^*. Briefly, mice were anaesthetized (4.5% chlorohydrate), the distal tendons of the TA and EDL muscles in both hindlimbs cut and attached to isometric force transducers (Dynamometer UFI Devices, UK), and the sciatic nerves exposed and sectioned. Isometric contractions were elicited by stimulating the nerve to the TA or EDL muscles using squarewave pulses of 0.02 ms duration at supra-maximal intensity. The number of motor units innervating the EDL muscle was determined by stimulating the motor nerve with stimuli of increasing intensity, resulting in incremental increases in twitch tension due to successive recruitment of motor axons with increasing threshold. The number of increments in twitch force was counted giving an estimate of the number of functional motor units present in muscle. The fatigue characteristics of EDL were examined by undertaking a fatigue test in which the EDL muscle was repeatedly stimulated at 40 Hz for 250 ms every second for 180 s. The resulting contractions were recorded on a pen recorder (Lectromed Multitrace 2) producing a fatigue trace from which an FI, a measure of muscle fatigability, can be determined by expressing the force at the end of the test as a ratio of the force at the start: FI = *F*_t180_/*F*_t0_. A muscle that is completely fatigue resistant has an FI approaching 1.0.

### Morphological assessment of muscle and neuronal tissue

For assessment of survival of motor neurons in the sciatic pool of the lumbar spinal cord (16,18), animals were perfused with saline followed with 4% paraformaldehyde (PFA) fixation. Spinal cords were removed, postfixed in 4% PFA and cryopreserved in 30% sucrose. Transverse sections (20 μm) of the lower L2–lower L6 lumbar region of fixed spinal cords cut on a cryostat serially onto glass slides and stained for Nissl (gallocyanin) (50). Nissl-stained motor neurons in the sciatic pool in every third section of the L3–L6 lumbar region, over 40 sections in total, were counted. Only large, polygonal neurons with a distinguishable nucleus and nucleolus and clearly identifiable Nissl structure were included in the counts. This method avoids the possibility of counting the same motor neuron in consecutive sections. At least five mice were analysed from each experimental group per time point.

For assessment of CSMNs, brains were removed intact from animals perfused as above, postfixed (4% PFA, overnight), and stored in PBS with sodium azide (0.01%) at 4°C. Brains were sectioned in 50 μm coronal planes and serially collected in 12-well plates. Sections mounted on Superfrost Plus glass slides (VWR) were stained for Nissl (cresyl violet). Antibodies used for assessment of CSMNs: anti-CTIP2 (1 : 1000; Abcam), anti-Cry-mu (1 : 500, Sigma), anti-Satb2 (1 : 1000; Abcam) and anti-LMO4 (1 : 500, Millipore). Sections for LMO4 were pretreated with 0.05% trypsin EDTA at 37°C for 10 min, followed by washes in blocking solution (0.5% BSA, 2% FBS, 0.1% Triton X-100 and 0.01% saponin, 0.02% sodium azide) and treatment with 50 mm ammonium chloride for 30 min. All sections were incubated with primary antibodies in blocking solution overnight at 4°C. Appropriate secondary antibodies (1 : 500 alkaline phosphatase conjugated, Santa Cruz Biotechnology; 1 : 500 Cy3-conjugated, Millipore) were applied. Sections with Cy3-conjugated antibodies were stained with DAPI (1 : 5000 in PBS) for 5 min. Sections for CTIP2 were processed with Vector blue AP substrate kit (Vector Laboratories) according to manufacturer's instructions. Sections were analysed using a Nikon Eclipse TE2000-E fluorescence microscope equipped with Intensilight C-HGFI (Nikon). Epifluorescent images were acquired using Digital Sight DS-Qi1MC CCD camera (Nikon). Three well-defined sections spanning the motor cortex (Bregma 0.86 mm, interaural 4.66 mm; Bregma 0.02 mm, interaural 3.82 mm; Bregma −1.22 mm, interaural 2.58 mm) (51) were used for quantitative analysis, as previously described (27,52).

Muscle histochemistry was performed on TA and EDL muscle sections by staining for the oxidative enzyme, as previously described (53). Briefly, serial cross-sections of fresh frozen TA and EDL were cut on a cryostat at 12 μm, collected on glass slides and stained for SDH activity to determine the oxidative capacity of the muscle fibres.

### Neuromuscular junction quantification

EDL muscles were dissected from mice of all three genotypes at 15 and 52 weeks of age. The numbers of whole EDL muscles analysed at 15 weeks of age: 7 WT, 4 *Sod1^+/D83G^* and 8 *Sod1^D83G/D83G^*. At 52 weeks: 11 WT, 8 *Sod1^+/D83G^* and 8 S*od1^D83G/D83G^*. Mice were perfused trans-cardial with saline solution and 4% PFA. Muscles were postfixed in 4% PFA and cryopreserved in sucrose. Whole EDL muscles were then embedded in OCT media (Tissue-Tek), frozen on dry-ice and stored at −80°C. A Bright™ cryostat was used to cut 20 µm longitudinal section of frozen EDL muscles which were subsequently collected onto poly-lysine-coated slides. Primary antibodies used: mouse monoclonal anti-synaptic vesicle (Developmental Studies Hybridoma Bank) Mouse monoclonal anti-neurofilament (165 kDa) (Developmental Studies Hybridoma Bank). M.O.M. biotinylated secondary antibody (Vector Labs) was used following manufacturer instructions. α-Bungarotxin-rhodamine was used to label postsynapsis and sections mounted. Images were acquired on a confocal microscope (Leica DFC 420C) and NMJ were manually counted.

### Immunocytochemistry

See Supplementary Material, Materials and Methods for immunocytochemistry experiments.

### qPCR, western blotting, dismutase activity and immunoprecipitations

qPCR, western blotting, dismutase activity and immunoprecipitation protocols are available in Supplementary Material, Materials and Methods.

### Embryonic motor neuron culture and mitochondrial membrane potential

Motor neuron culture and measurement of mitochondrial membrane potential are described in Supplementary Material, Materials and Methods.

### Statistical analysis

An ANOVA test was used to compare between WT, *Sod1^+/D83G^* and *Sod1^D83G/D83G^*genotypes per time point followed by Bonferroni's multiple comparisons testing correction for weight, dismutase activity, protein quantification, Echo MRI analysis, grip strength, rotarod, startle response and sensory analysis. Wheel running behaviour was analysed using a repeated measures ANOVA. Cox survival analysis was used to analyse SHIRPA and survival data. The Mann–Whitney test was used to compare between genotypes per time point or *Sod1^D83G/D83G^* cohorts across time points for TA and EDL maximum muscle force, surviving motor units, FI and motor neuron survival. Two-tailed tests were used in all instances and significance level was set at *P* < 0.05.

## Supplementary Material

Supplementary Material is available at *HMG* online.

## Funding

This work was supported by the Motor Neurone Disease Association (A.A., E.F.), Packard Center for ALS at Johns Hopkins (A.A., E.F., L.G.), Wellcome Trust (DLHB, 095698/Z/11/Z), Medical Research Council (E.F., A.A.) and Les Turner ALS Foundation (P.H.O.). L.G. and E.F. are funded by the European Community's Seventh Framework Programme (FP7/2007-2013). L.G. is the Graham Watts Senior Research Fellow, funded by The Brain Research Trust. Funding to pay the Open Access publication charges for this article was provided by the Medical Research Council.

## Supplementary Material

Supplementary Data
