# Supplementary material for: A novel SOD1-ALS mutation separates central and peripheral effects of mutant SOD1 toxicity
Source: Hum Mol Genet. 2014 Dec 2;24(7):1883–97. doi: 10.1093/hmg/ddu605 (PMC4355022; doi:10.1093/hmg/ddu605)
Supplement: Supplementary Data [file supp_24_7_1883__index.html]

A novel SOD1-ALS mutation separates central and peripheral effects of mutant SOD1 toxicity — A novel SOD1-ALS mutation separates central and peripheral effects of mutant SOD1 toxicity — Supplementary Data 

# A novel SOD1-ALS mutation separates central and peripheral effects of mutant SOD1 toxicity

## Supplementary Data

Supplementary Data

**Files in this Data Supplement:**

- Supplementary Data - Docx file
- Supplementary Figure 1 - tif file
- Supplementary Figure 2 - tif file
- Supplementary Figure 3 - tif file
- Supplementary Figure 4 - tif file
- Supplementary Figure 5 - tif file
- Supplementary Figure 6 - jpg file
- Supplementary Figure 7 - tif file
- Supplementary legends - docx file
- Supplementary Video 1 - mp4 file
