# Supplementary material for: A novel SOD1-ALS mutation separates central and peripheral effects of mutant SOD1 toxicity
Source: Hum Mol Genet. 2014 Dec 2;24(7):1883–97. doi: 10.1093/hmg/ddu605 (PMC4355022; doi:10.1093/hmg/ddu605)
Supplement: Supplementary Data [file supp_ddu605_ddu605supp.docx]

**Supplementary Materials and Methods**

**A novel SOD1-ALS mutation separates central and peripheral effects of mutant SOD1 toxicity**

**Peter I Joyce^1^, Philip McGoldrick^2#^, Rachele A Saccon^2#^, William Weber^3#^, Pietro Fratta^2^, Steven J West^4^, Ning Zhu^4^, Sarah Carter^1^, Vinaya Phatak^1^, Michelle Stewart^1^, Michelle Simon^1^, Saumya Kumar^1^, Ines Heise^1^, Virginie Bros-Facer^2^, James Dick^2^, Tu Vinh Luong^5^, Patrick M Nolan^1^, Timothy Meyer^6^, Sebastian Brandner^2^, David LH Bennett^4^, P Hande Ozdinler^3,7,8^, Linda Greensmith^2,^* , Elizabeth M C Fisher^2,^*, Abraham Acevedo-Arozena^1,^*.**

^1^ MRC Mammalian Genetics Unit, Harwell, Oxfordshire, OX11 0RD, UK

^2^ UCL Institute of Neurology, MRC Centre for Neuromuscular Disease, Queen Square, London, WC1N 3BG, UK

^3^ Les Turner ALS Laboratory II, Department of Neurology, Northwestern University, Feinberg School of Medicine, Chicago IL, 60611, USA

^4^ Nuffield Department of Clinical Neurosciences, University of Oxford, Oxford, OX3 9DU, UK

^5^ Department of Cellular Pathology, Royal Free London NHS Foundation Trust, Pond Street, London, NW3 2QG, UK

^6^ UCL Cancer Institute, Paul O'Gorman Building, 72 Huntley Street, London, WC1E 6BT, UK

^7^ Robert H. Lurie Cancer Center, Northwestern University, Chicago IL, 60611, USA

^8^ Cognitive Neurology and Alzheimer’s Disease Center, Northwestern University, Chicago IL, 60611, USA

# Equal contributors

* Corresponding authors:

* Abraham Acevedo-Arozena, Medical Research Council Mammalian Genetics Unit, Harwell, Oxfordshire, OX11 0RD, UK. [a.acevedo@har.mrc.ac.uk](mailto:a.acevedo@har.mrc.ac.uk)

* Elizabeth M C Fisher, Institute of Neurology and MRC Centre for Neuromuscular Disease, UCL, Queen Square, London, WC1N 3BG, UK. [e.fisher@prion.ucl.ac.uk](mailto:e.fisher@prion.ucl.ac.uk)

* Linda Greensmith, Institute of Neurology and MRC Centre for Neuromuscular Disease, UCL, Queen Square, London, WC1N 3BG, UK. [l.greensmith@ucl.ac.uk](file:///C:\Users\a.acevedo\AppData\Roaming\Microsoft\Word\l.greensmith@ucl.ac.uk)

**Sequencing of the *Sod1^D83G^* founder mouse**

Genomic DNA from the founder *Sod1 D83G* mouse (F1) was sequenced using the Illumina platform (HiSeq2000) and the paired-end reads were aligned to the mouse reference genome (mm9) using Burrow-Wheeler Aligner (BWA) ([Li and Durbin, 2010](#_ENREF_52)). SNP detection was performed on chromosome 16 using a combination of Samtools ([Li et al., 2009](#_ENREF_51)) and Genome Analysis Toolkit (GATK) ([DePristo et al., 2011](#_ENREF_17)). SNPs in the resulting formatted file (variant call format (VCF)) were filtered if their quality scores were < 100, allele frequency was out of range (0.4 - 0.7) and read depth was < 3. Sites in the VCF file were further filtered by removing strain specific SNPs found in the Mouse Genomes Project ([Keane et al., 2011](#_ENREF_45)). The remaining novel SNPs were annotated using NGS-SNP ([Grant et al., 2011](#_ENREF_28)) and the coding SNPs validated by conventional Sanger sequencing/PryoSequencing. The closest coding SNP was ~46 mb proximal to the *Sod1* gene and did not segregate with lifespan or weight abnormalities in *Sod1^D83G/D83G^* mice.

**Behavioural Analysis**

Phenotyping cohort sizes for grip strength, rotarod and startle response were as follows: female: 11 WT, 13 *Sod1^+/D83G^*, 11 *Sod1^D83G/D83G^*; male: 12 WT, 14 *Sod1^+/D83G^*, 11 *Sod1^D83G/D83G^*; Grip strength (BioSeb, Chaville, France) from all four limbs was taken twice each every four weeks, from 6-weeks of age to the humane endpoint, and values averaged for each time point per animal. Animals were subjected to an accelerating rotarod (Ugo Basile) twice a day, three times a week, from 7 weeks of age.

Modified SHIRPA methods were performed as described previously ([Rogers et al., 1997](#_ENREF_73); [Rogers et al., 2001](#_ENREF_74)). Tremors were qualitatively assessed by observation in a viewing jar and recorded as having no tremors (0), mild tremors (1), moderate tremors (2) or severe tremors (3). Pelvic elevation was assessed as normal (2) or reduced (1). Negative geotaxis involves animals walking down a vertical grate. Animals were scored as able to move down the grate without any problems (0) or slipping at points down the grate (1). Mice were subject to modified SHIRPA analysis from 6 weeks of age until they reached their humane endpoint. Cohort sizes were as follows: female: 13 WT, 14 *Sod1^+/D83G^*, 12 *Sod1^D83G/D83G^*; male: 12 WT, 15 *Sod1^+/D83G^*, 11 *Sod1^D83G/D83G^*.

Monitoring of in-cage wheel running activity was performed for 7 days using the PhenoMaster (TSE systems) system. Recordings are calculated from the first hour of the night when animals are most active, using PhenoMaster automated data software. 7 *Sod1^+/D83G^* and 7 WT littermates were analysed at 44 and 88 weeks of age.

WT, *Sod1^+/D83G^*and *Sod1^D83G/D83G^* littermates were assessed for sensory abnormalities using Von Frey, Cold Plate, Hargreaves and Randall-Selitto Tests. Animals acclimatized 30-60 minutes prior to testing of both paws. Following training sessions sensory thresholds to a number of different sensory modalities were assessed, as previously described ([Fricker et al., 2009](#_ENREF_29)). The experimenter was blind to genotype. (1) Mice were tested for 50% paw withdrawal threshold to static mechanical stimuli using von Frey filaments (between 0.98 and 19.61 mN).  Briefly, animals were placed in plastic chambers resting on wire mesh, allowing an initial stimulation of 5.88 mN to the plantar surface of each hind paw, proximal to the toe pads.  Further testing followed the up-down method, as previously described ([Chaplan et al., 1994](#_ENREF_16)). (2) Cold Plate Test (cold nociceptive sensitivity). Animals were placed onto a stainless steel plate, surrounded by clear plexiglass (4"x8"x6"), with a constant plate temperature of 0°C ± 0.5°C.  Pain-related behaviours (licking, biting or lifting of the hind paws and jumping) were quantified over 5 min, and total number of pain-related behaviours recorded, as previously described ([Bennett and Xie, 1988](#_ENREF_7); [Jasmin et al., 1998](#_ENREF_40)). The sum of two tests was taken as a measure of baseline cold nociceptive sensitivity. (3) Reflex withdrawal to a noxious thermal stimuls was assessed using Hargreaves apparatus. Animals were placed into plastic chambers rested on a glass plate, allowing efficient and even conduction of heat. Latency of withdrawal from a heat stimulus (infrared source) was recorded three times from each hind paws, with at least 1 min between recordings on the same paw, as described previously ([Hargreaves et al., 1988](#_ENREF_36)). Mean values across three tests on both paws on each test day were used to derive the heat nociceptive withdrawal threshold for each animal. (4) Randall-Selitto Test (mechanical pressure threshold). Mechanical nociceptive pressure tolerance to the hind paws was assessed using an electromechanical algesimeter, and measuring hind paw withdrawal thresholds to increasing pressure ([Takesue et al., 1969](#_ENREF_81)).  The initial load applied to the paw was 0 mN, and increased linearly to a maximum force of 1472 mN (150 g).  Upon withdrawal, pressure was released, and force of withdrawal noted. Mean values of three tests were taken as the nociceptive threshold to mechanical pressure stimulation.

**Echo MRI analysis**

WT, Sod1+/D83G and Sod1D83G/D83G littermates were assessed for whole body lean and fat mass using Echo MRI as per the manufacturer’s instructions (Echo Medical Systems). Echo MRI was performed at 6, 35, 52 and 88 weeks of age for female genotypes, 52 and 88 weeks for male genotypes, both on a C57BL/6J background; values were averaged for each time point and genotype. Cohort sizes per time point were as follows: 6 weeks of age female: 5 WT, 5 *Sod1^+/D83G^*, 5 *Sod1^D83G/D83G^*; 35 weeks female: 13 WT, 14 *Sod1^+/D83G^*, 6 *Sod1^D83G/D83G^*; 52 weeks of age female: 13 WT, 14 *Sod1^+/D83G^*, 8 *Sod1^D83G/D83G^*; 88 weeks female: 9 WT, 13 *Sod1^+/D83G^*; 52 weeks male: 12 WT, 16 *Sod1^+/D83G^*, 9 *Sod1^D83G/D83G^* , 88 weeks male: 5 WT, 5 *Sod1^+/D83G^*.

**Immunocytochemistry**

Transverse sections (20 μm) from the lumbar region of fixed spinal cords were immuno-fluorescently stained with anti-GFAP (Cy-conjugated mouse monoclonal, Sigma; used at 1:1000) and anti-IBA1 (rabbit polyclonal, Abcam; used at 1:500), visualised using a secondary AlexaFluor488-conjugated antibody (Invitrogen) and counterstained with NeuroTrace® 435⁄455 fluorescent Nissl stain (Invitrogen). A C-terminal p62 antibody (Progen, Germany; 1:300), ubiquitin antibody (Dako UK; 1 : 1000), USOD antibody (kind gift of Avi Chakrabartty and Janice Robertson, University of Toronto, Canada; 0.3 μg/ml), SEDI antibody (kind gift from Janice Robertson, University of Toronto, Canada; 1:1000), D3H5 (kind gift from Jean-Pierre Julien, Universite´ Laval, Canada; 1:1000) were used to stain both PFA fixed lumbar spinal cord sections and formalin fixed, paraffin embedded spinal cord sections from *Sod1^D83G^* mice and *SOD1^D83G^* human samples. Paraffin embedded sections, once incubated with appropriate secondary antibodies, were developed using 3,30-diaminobenzidine and counterstained with hematoxylin. Confocal images were taken using a Zeiss 710 microscope.

**qPCR**

RNA was isolated from brain using a RNeasy kit (Qiagen). cDNA was produced from 1.5 μg of DNAse treated mRNA, using Taqman Reverse Transcription Reagents (Applied Biosystems). *Sod1* qPCR was performed in triplicate from three 9 week old male WT, *Sod1^+/D83G^* and *Sod1^D83G/D83G^* littermates in 20 μl reactions using Fast SYBR® Green Master Mix (Applied Biosystems) and analysed using Fast System SDS software (Applied Biosystems); PCR products were verified by agarose gel electrophoresis. Gene expression data for *Sod1^+/D83G^* and *Sod1^D83G/D83G^* brains were analysed using the ΔΔCT method and normalized using S16 and GAPDH endogenous reference genes relative to WT spinal cord ([Livak and Schmittgen, 2001](#_ENREF_49)). Primers were designed to span exon-exon boundaries and were as follows: *Sod1*, PJ-309 TACACAAGGCTGTACCAGTG, PJ-310 CCAACATGCCTCTCTTCATC; 16S, 16s-F TTCTGGGCAAGGAGCGATT, 16s-R GATGGACTGTCGGATGGCA; GAPDH, Gapdh-F ACGGGAAGCTCACTGGCATGGCCTT, Gapdh-R CATGAGGTCCACCACCCGTTGCTG.

Quantitative pyrosequencer analysis of cDNA from brains of 9-week old male *Sod1^+/D83G^* samples (n = 3) was used to compare WT and mutant *Sod1^D83G^* mRNA levels by quantifying the area under the curve of traces obtained from pyrosequencer reactions using a PSQ8HS96 pyrosequencer (Biotage). Average levels of allele expression were WT (A = 50.7%), mutant (G = 49.3%) (p = 0.223).

**Western blotting and dismutase activity assay**

Snap frozen spinal cord tissue from male 9 week and 65 week old WT, *Sod1^+/D83G^* and *Sod1^D83G/D83G^* littermate mice were homogenised in RIPA buffer (150 mM NaCl, 1% NP40, 0.5% Na deoxycholate, 0.1% SDS, 50 mM Tris pH 7.5) with protease inhibitors (Roche Diagnostic) using lysing matrix tubes D (MP Biomedicals, Germany) and a Fast-Prep-24 homogenizer at 4°C. Homogenates were centrifuged at 13,000 rpm for 15 min, soluble fraction removed and insoluble fraction mixed with 6 M urea, 50 mM Tris-HCl, 1 mM DTT, 150 mM NaCl solution, re-solubilised using a micro-tip sonicator (Soniprep 150 Plus, MSE, UK) and centrifuged for 15 min at 13,000 rpm. 20 μg of soluble and insoluble fractions were resolved by SDS–PAGE (NUPAGE system, Invitrogen) and transferred to low fluorescent PVDF (LF-PVDF) membranes (Millipore). Blots were incubated with primary antibodies at the following concentrations: anti-SOD1 (Enzo life sciences), 1:1000; anti-α tubulin (Abcam), 1:5000; anti-actin (Sigma), 1:1000. Protein was visualized using anti-mouse or anti-rabbit secondary antibodies IRDye^®^ (Li-Cor Biosciences) at 1:5000 dilution and quantified using the Odyssey imaging system (Li-Cor Biosciences).

SOD1 dismutase activity: 30 μg of the soluble fraction of brain homogenates (see spinal cord extraction above) from WT, *Sod1^+/D83G^*and *Sod1^D83G/D83G^* (n = 3 per genotype) were resolved by native PAGE 10% (NUPAGE system, Invitrogen), incubated for 45 minutes in nitroblue treazolium (NBT) solution (100 ml: 15 mg NBT, 10 mg riboflavin, 680.45 mg KPO_4_ (pH 7.8), 10 μl TEMED) in the dark, then exposed to a light box. SOD activity appears colourless against a blue background.

**Immunoprecipitations**

Frozen spinal cords from WT, *Sod1^+/D83G^* and *Sod1^D83G/D83G^* littermates were homogenised as above. 2 mg of soluble protein was incubated with 2 μg of USOD, SEDI or D3H5 antibody for 24 hours at 4°C, mixed with 50 μl of 50% (w/v) Protein G Sepharose beads (Sigma) overnight at 4°C, then immunoprecipitates eluted with SDS sample buffer and analysed with SDS PAGE and western blotting. USOD and SEDI successfully immunoprecipitated misfolded SOD1 protein from *SOD1^G93A^* extracts.

**Embryonic motor neuron culture**

Motor neuron cultures were prepared as previously described ([Bilsland et al., 2008](#_ENREF_8)). Briefly, embryonic spinal cords (E13.5) were removed, ventral horns isolated, motor neurons seeded at 40,000 cells/cm^2^ and maintained in complete neurobasal medium (CNB; 200 ml of which contains:  191 ml neurobasal medium, 4 ml B27 supplement (1 unit/ml; Gibco), 4 ml horse serum (PAA Laboratories), 500 µl 0.5 mM l-glutamine (Gibco), 100 µl 0.05% 2-mercaptoethanol (Gibco), 20 µl ciliarly neurotrophic factor (CNTF; 500 pg/ml; Alomone Labs), 2 µl glial-derived neurotrophic factor (GDNF; 100 pg/ml; Alomone Labs), 2 µl brain-derived neurotrophic factor (BDNF; 100 pg/ml),  2 ml P/S, supplemented with 0.02 mg/ml DNase, in a 37°C, 5% CO_2_ humidified incubator for a minimum of 7 days.

**Mitochondrial membrane potential**

At 7 days in culture embryonic motor neurons with at least 3 processes were loaded for 40 min at room temperature with 20 nM tetramethylrhodamine methylester (TMRM) (Molecular Probes, Eugene, OR) in a HEPES-buffered salt solution (HBSS) composed: 156 NaCl mM, 3 KCl mM, 2MgSO_4_ mM, 1.25 KH_2_PO_4_ mM, 2 CaCl_2_ mM, 10 mM glucose and 10 mM Hepes, pH to 7.35. TMRM measurements were made using a Zeiss LSM 510 equipped with a META detection system and a 40x oil immersion objective. Illumination intensity was kept to a minimum (at 0.1-0.2 % of laser output) to avoid phototoxicity and pinhole set to give an optical slice of ~2 μm. TMRM was excited using the 565 nm laser line and fluorescence measured above 580 nm. All data presented were obtained from at least 3 coverslips and 2-3 different cell preparations.
