# Supplementary figures and images for: A novel SOD1-ALS mutation separates central and peripheral effects of mutant SOD1 toxicity
Source: Hum Mol Genet. 2014 Dec 2;24(7):1883–97. doi: 10.1093/hmg/ddu605 (PMC4355022; doi:10.1093/hmg/ddu605)

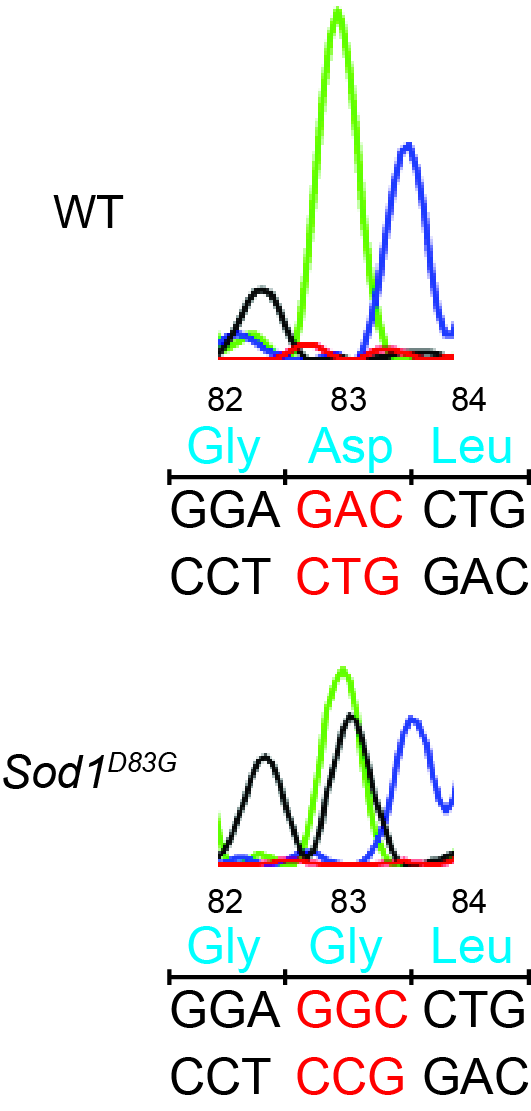

Supplement: Supplementary Data [file supp_ddu605_ddu605supp_fig1.tif]

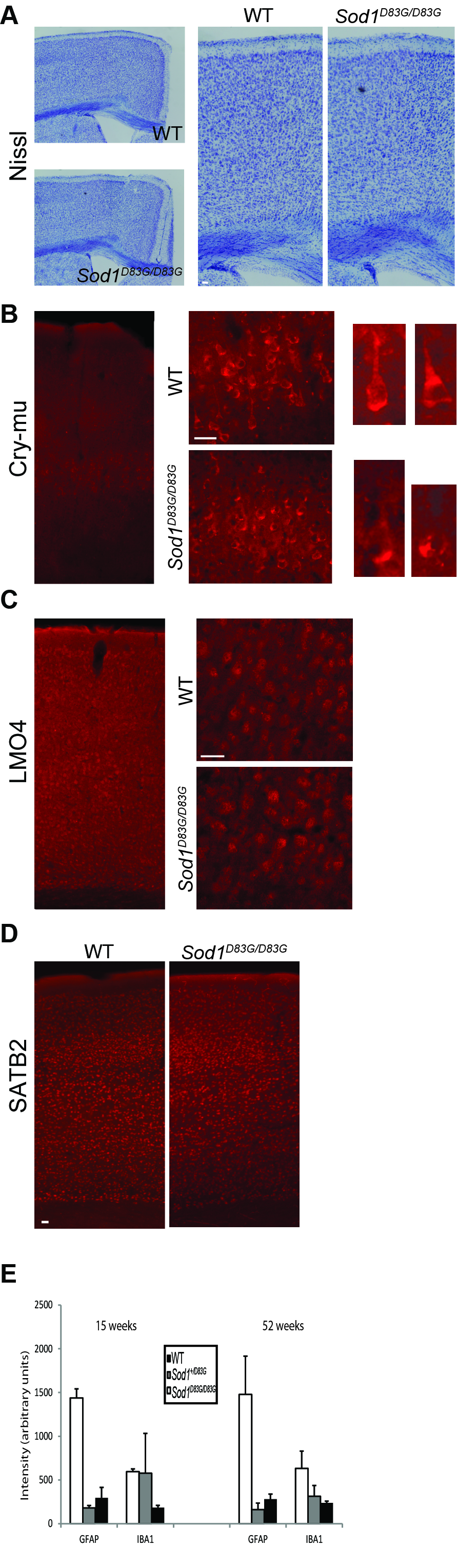

Supplement: Supplementary Data [file supp_ddu605_ddu605supp_fig2.tif]

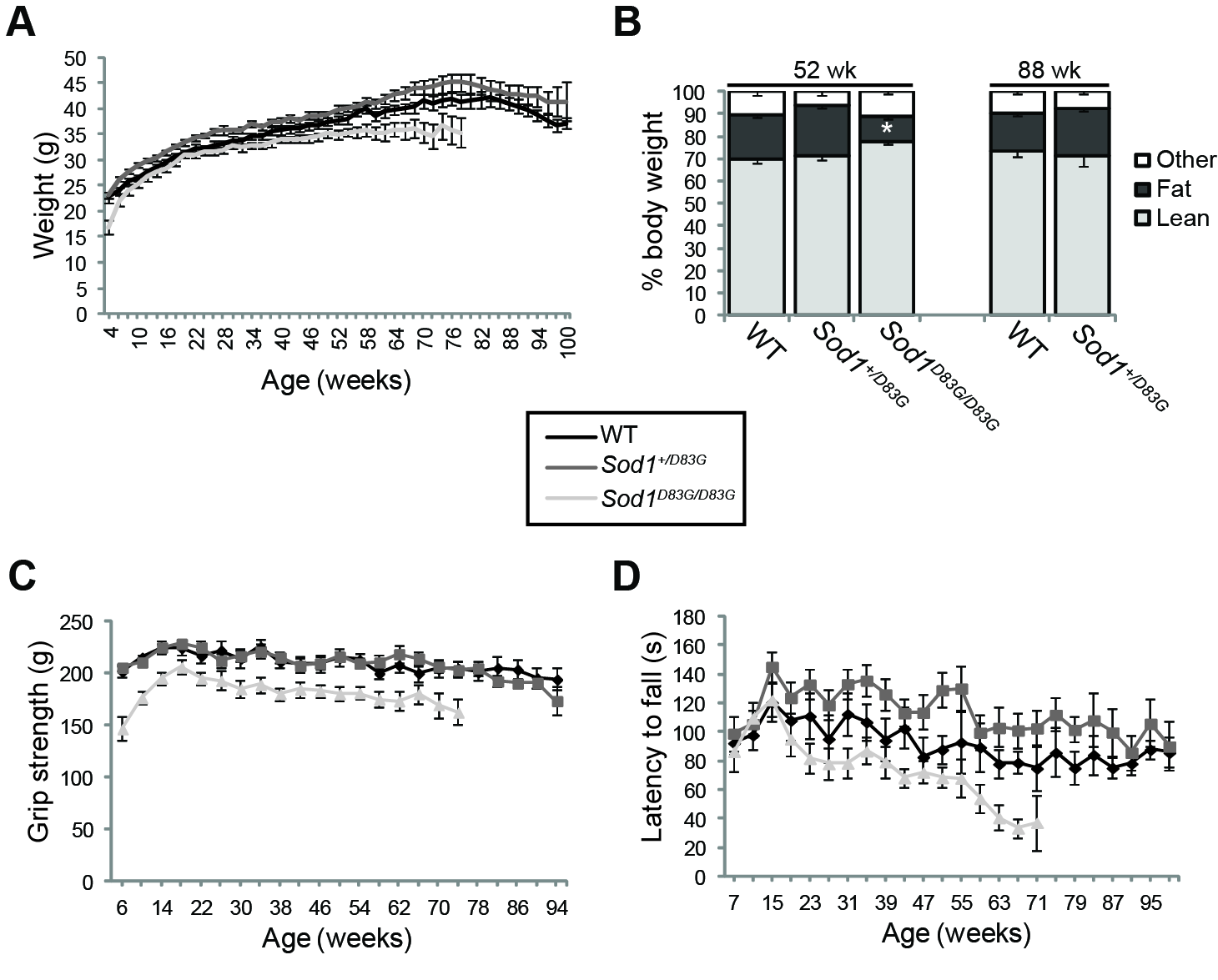

Supplement: Supplementary Data [file supp_ddu605_ddu605supp_fig3.tif]

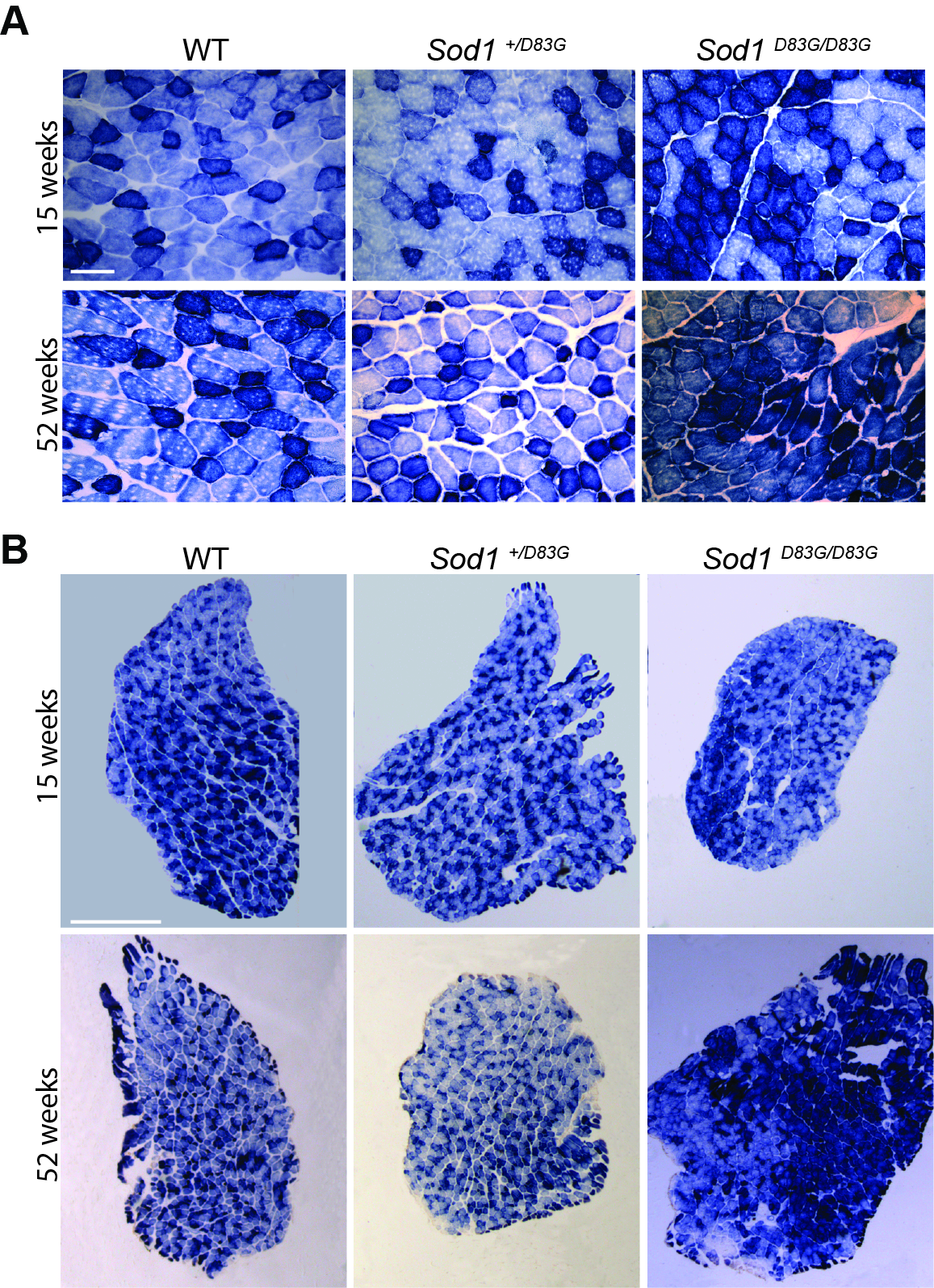

Supplement: Supplementary Data [file supp_ddu605_ddu605supp_fig4.tif]

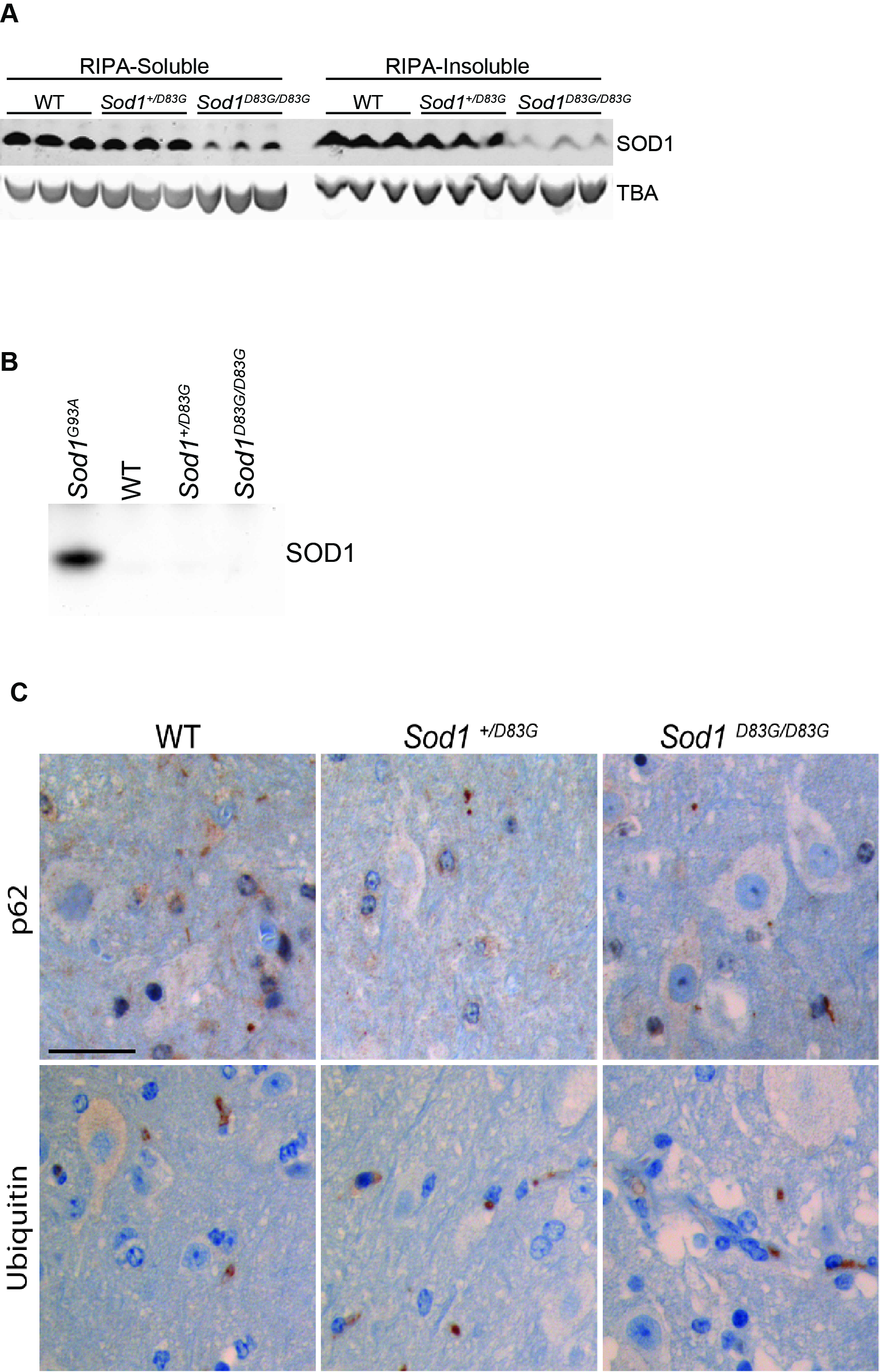

Supplement: Supplementary Data [file supp_ddu605_ddu605supp_fig5.tif]

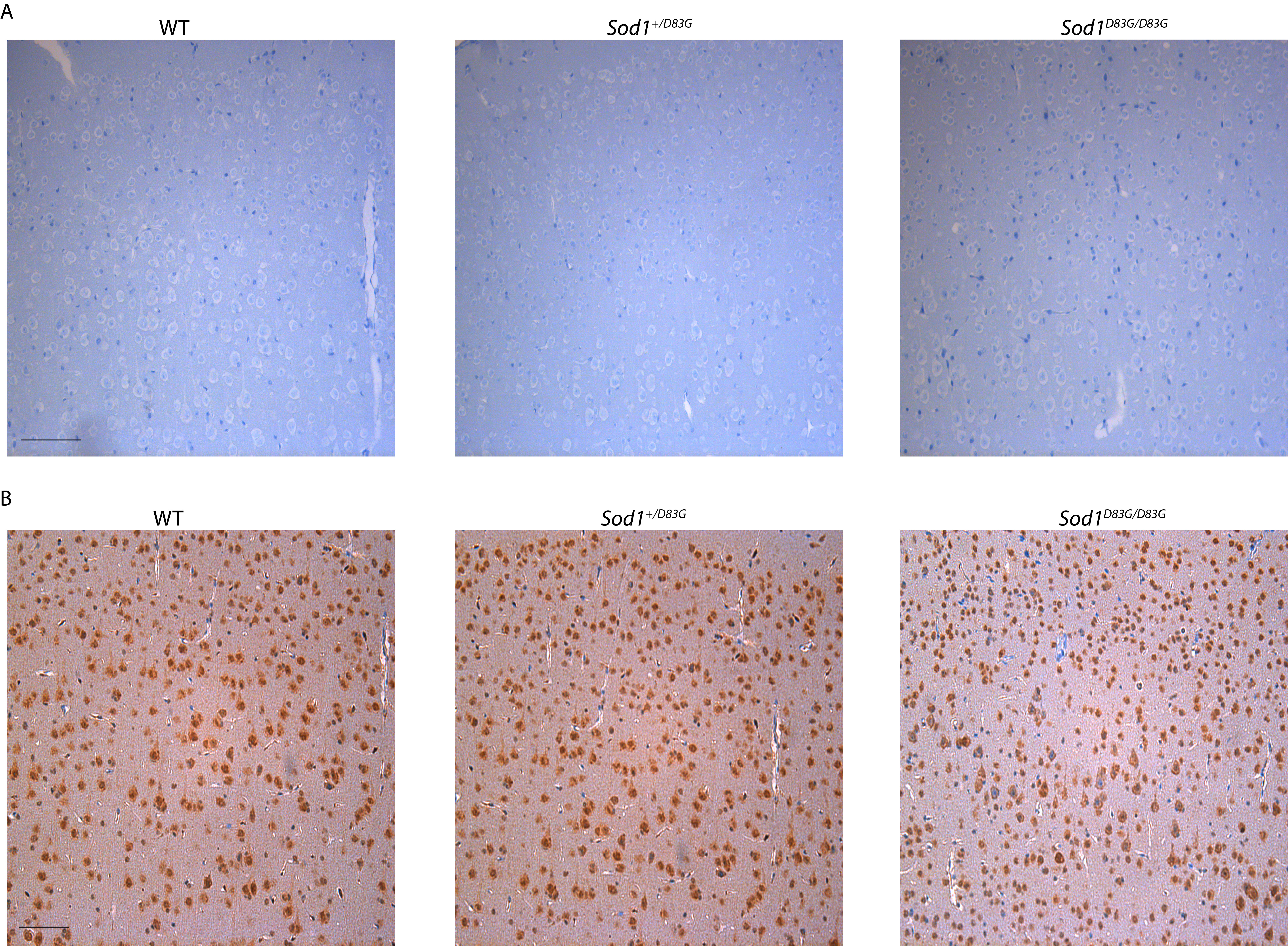

Supplement: Supplementary Data [file supp_ddu605_ddu605supp_fig6.jpg]

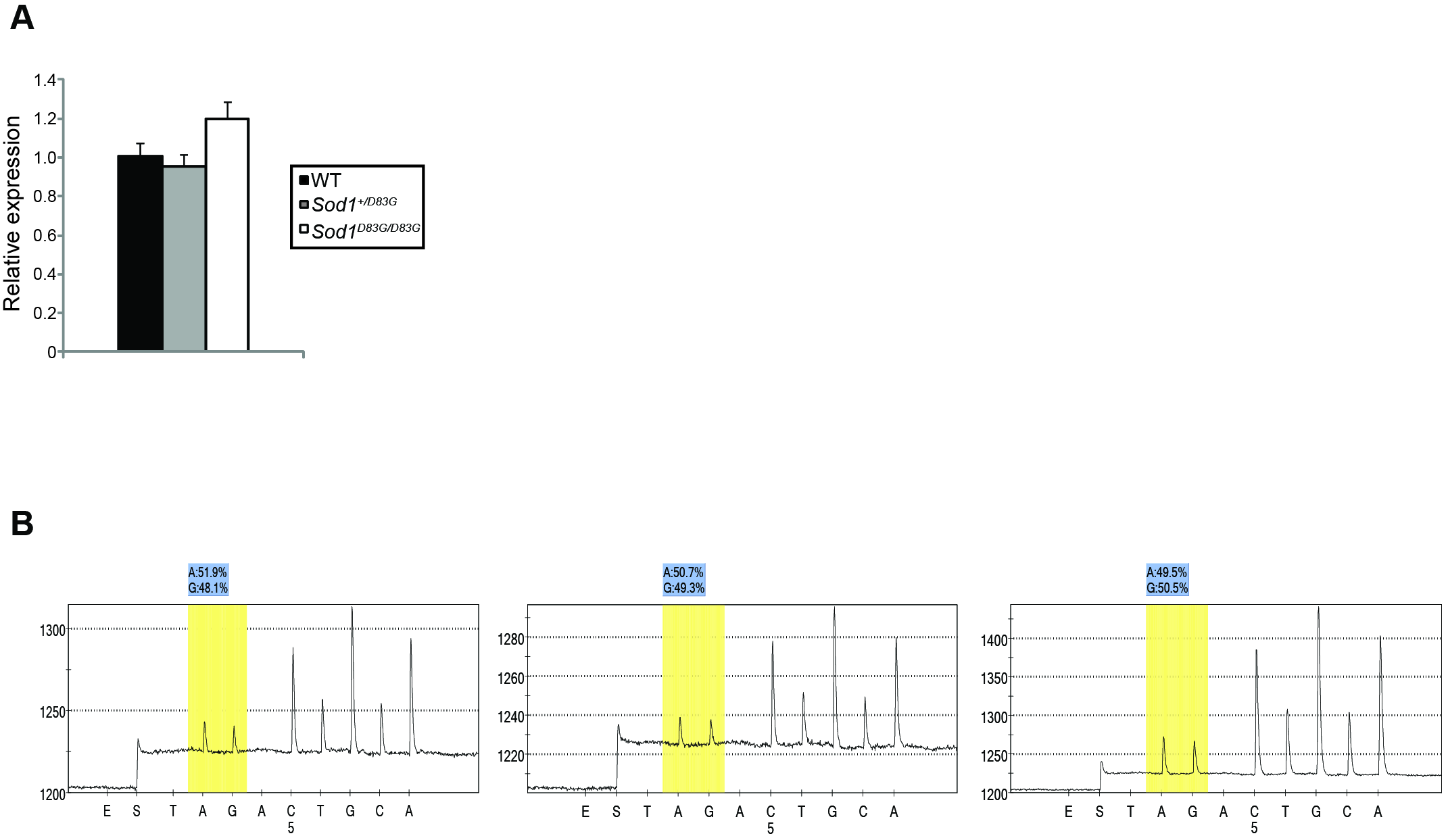

Supplement: Supplementary Data [file supp_ddu605_ddu605supp_fig7.tif]
