# Supplementary material for: A novel SOD1-ALS mutation separates central and peripheral effects of mutant SOD1 toxicity
Source: Hum Mol Genet. 2014 Dec 2;24(7):1883–97. doi: 10.1093/hmg/ddu605 (PMC4355022; doi:10.1093/hmg/ddu605)
Supplement: Supplementary Data [file supp_ddu605_ddu605supp_legends.docx]

**Supporting Information**

**Figure S1.** Identification of the *Sod1^D83G^* mutation. Sequence traces of part of exon 4 of *Sod1*. The mutation is from A to G and seen as a double peak in a *Sod1^+/D83G^* heterozygote, causing a non-conservative amino acid change from aspartic acid to glycine.

**Figure S2.** Degeneration of UMNs is specific to the corticospinal motor neurons in *Sod1^D83G/D83G^* mice. All mice are 29 weeks of age. (A) Nissl staining of the cerebral cortex reveals no differences between genotypes; there is no aberrant defect in cortical layers. Motor cortex is enlarged in the right-hand image. (B) Cry-mu expression restricted to corticospinal motor neurons (CSMNs) within layer V of the motor cortex, suggests differences between WT and *Sod1^D83G/D83G^* CSMNs. (C) LMO4 expression, excluded from CSMNs and detected in CPNs within layer V, shows no differences between WT and *Sod1^D83G/D83G^* littermates. (D) SATB2 expression in the motor cortex of WT and *Sod1^D83G/D83G^* littermates. SATB2, which is excluded from corticospinal motor neurons (CSMN) and is mainly expressed by callosal projection neurons (CPN) that are present throughout the cortex, is comparable between WT and *Sod1^D83G/D83G^* littermates. All scale bars are 50 μm. (E) GFAP and IBA1 quantification from Figure 1C. At least two images per genotype were quantified using Volocity at both time points. GFAP intensity is significantly elevated in *Sod1^D83G/D83G^* when compared to WT (15 weeks: p= 0.003; 52 weeks: p= 0.013). IBA1 was also elevated in *Sod1^D83G/D83G^* when compared to WT, but differences did not reach statistical significance (15 weeks: p= 0.65; 52 weeks: p= 0.06). Not significant differences appeared at any time point between WT and *Sod1^+/D83G^* mice.

**Figure S3.** Body mass, behavioural and motor deficits in male *Sod1^D83G/D83G^* mice. (A) Weights for male mice from 4 weeks to humane endpoint; cohort sizes for (A, C and D) started as follows: 12 WT, 14 *Sod1^+/D83G^*, 11 *Sod1^D83G/D83G^*. Due to death of mice with age at least 5 mice were assessed per genotype at later time points. Weight is reduced in *Sod1^D83G/D83G^* mice from 4-weeks (p = 0.034) (B) Echo MRI assessment of lean and fat mass for males at 52 and 88 weeks of age. Fat mass is reduced in 52-week old *Sod1^D83G/D83G^* mice (11% ± 1%) compared to WT littermates (20% ± 2%) (*, p = 0.004). (C) Grip strength from 6 weeks to humane endpoint. Grip strength is reduced in *Sod1^D83/D83G^* mice compared to WT littermates (p < 0.05). (D) Rotarod (latency to fall) from 7 weeks to humane endpoint. Rotarod performance deteriorates in *Sod1^D83G/D83G^* mice compared to WT littermates (67-weeks, p < 0.05). Numbers for (A-D) represent the mean ± SEM (*, p < 0.05).

**Figure S4.** Muscle histology of *Sod1^D83G/D83G^* mice. SDH staining of (A) TA and (B) EDL cross sections from 15- and 52-week old mice. (A) TA of 15 week *Sod1^D83G/D83G^* mice has more intensely stained, type I muscle fibres; the number of intensely stained fibres increases further in 52-week *Sod1^D83G/D83G^* mice. Scale bar is 100 μm. (B) EDL of 15-week *Sod1^D83G/D83G^* mice appears similar to WT and *Sod1^+/D83G^* EDLs, whilst EDL from 52-week old *Sod1^D83G/D83G^* mice display a greater number of intensely stained type I muscle. Scale bar is 500 μm.

**Figure S5.** (A) SOD1 D83G protein levels are unchanged between RIPA soluble and insoluble fractions. Representative immunoblot analysis of SOD1 protein levels in soluble and insoluble fractions from male 65-week old WT, *Sod1^+/D83G^* and *Sod1^D83G/D83G^* spinal cords (n = 3 per genotype). α-tubulin (TBA) provides a protein loading reference. (B) Misfolded SOD1 antibody SEDI is unable to immunoprecipitate misfolded SOD1 from *Sod1^D83G^* mice. Immunoprecipitations were performed on spinal cord lysates from WT, *Sod1^+/D83G^* and *Sod1^D83G/D83G^* mice and western blots probed with anti-SOD1; spinal cord lysates from *SOD1^G93A^* mice were used as a positive control. (C) p62 and ubiquitin staining of the spinal cord from WT, Sod1^+/D83G^ and Sod1^D83G/D83G^ mice at 52 weeks of age does not reveal any differences in inclusion pathology. Scale bar is 20 μm.

**Figure S6.** (A) Immunohistochemistry with the SED1 antibody does not reveal misfolded SOD1 at 52 weeks from spinal cord. Scale bar is 100 μm. (B) Ubiquitin staining from cortex of 52 weeks old mice does not reveal any pathology. Scale bar is 100 μm.

**Figure S7**. (A) Sod1 mRNA expression levels do not differ between WT, *Sod1^+/D83G^* and *Sod1^D83G/D83G^* littermates. Analysis of *Sod1* expression levels in spinal cord of 6-week old male WT, *Sod1^+/D83G^* and *Sod1^D83G/D83G^* littermates (n = 3 per genotype); expression in *Sod1^+/D83G^* and *Sod1^D83G/D83G^* mice is normalised to *Sod1* expression in WT, taken as a value of 1. (B) *Sod1* allele expression analysed via quantitative pyrosequencing from *Sod1^+/D83G^* spinal cords. RNA was extracted from spinal cords of 3 *Sod1^+/D83G^* mice, cDNA produced and allele proportion assessed via pyrosequencing. *Sod1^+/+^* and *Sod1^D83G/D83G^* were used as controls, with WT allele [A] being solely produced in *Sod1^+/+^* and mutant allele [G] only expressed in *Sod1^D83G/D83G^* spinal cords. In *Sod1^+/D83G^* spinal cords, where both alleles are expressed, similar proportions of WT and mutant alleles are expressed. Percentage of WT allele [A]: 50.7 ± 1.2. Percentage of mutant allele [G]: 49.3 ± 1.2. p=0.226.

**Video S1.** Progressive motor deficits of *Sod1^D83G/D83G^* mice. A *Sod1^D83G/D83G^* mouse at 6, 30, 52 and 83 weeks of age showing age dependent development of tremors, gait abnormalities, reduced pelvic elevation, and overall poor health.

**Table S1.** Ratios of inheritance for *Sod1^D83G^* mice

**Observed ratios**

| **Genotype** | **female** | **male** | **combined** |
| --- | --- | --- | --- |
| *Sod1^+/+^* | 82 | 85 | 167 |
| *Sod1^+/D83G^* | 186 | 176 | 362 |
| *Sod1^D83G/D83G^* | 43** | 58* | 101** |

Significance of difference by comparison to Mendelian ratios (1:2:1) (* p = 0.018; ** p < 0.001).
